# Supplementary material for: Initiation of Quality Control during Poly(A) Translation Requires Site-Specific Ribosome Ubiquitination
Source: Mol Cell. 2017 Feb 16;65(4):743–750.e4. doi: 10.1016/j.molcel.2016.11.039 (PMC5316413; doi:10.1016/j.molcel.2016.11.039)
Supplement: Document S1. Figures S1–S4 [file mmc1.pdf]

Molecular Cell, Volume *65*

Supplemental Information

Initiation of Quality Control during Poly(A) Translation Requires Site-Specific Ribosome Ubiquitination

Szymon Juszkiewicz and Ramanujan S. Hegde

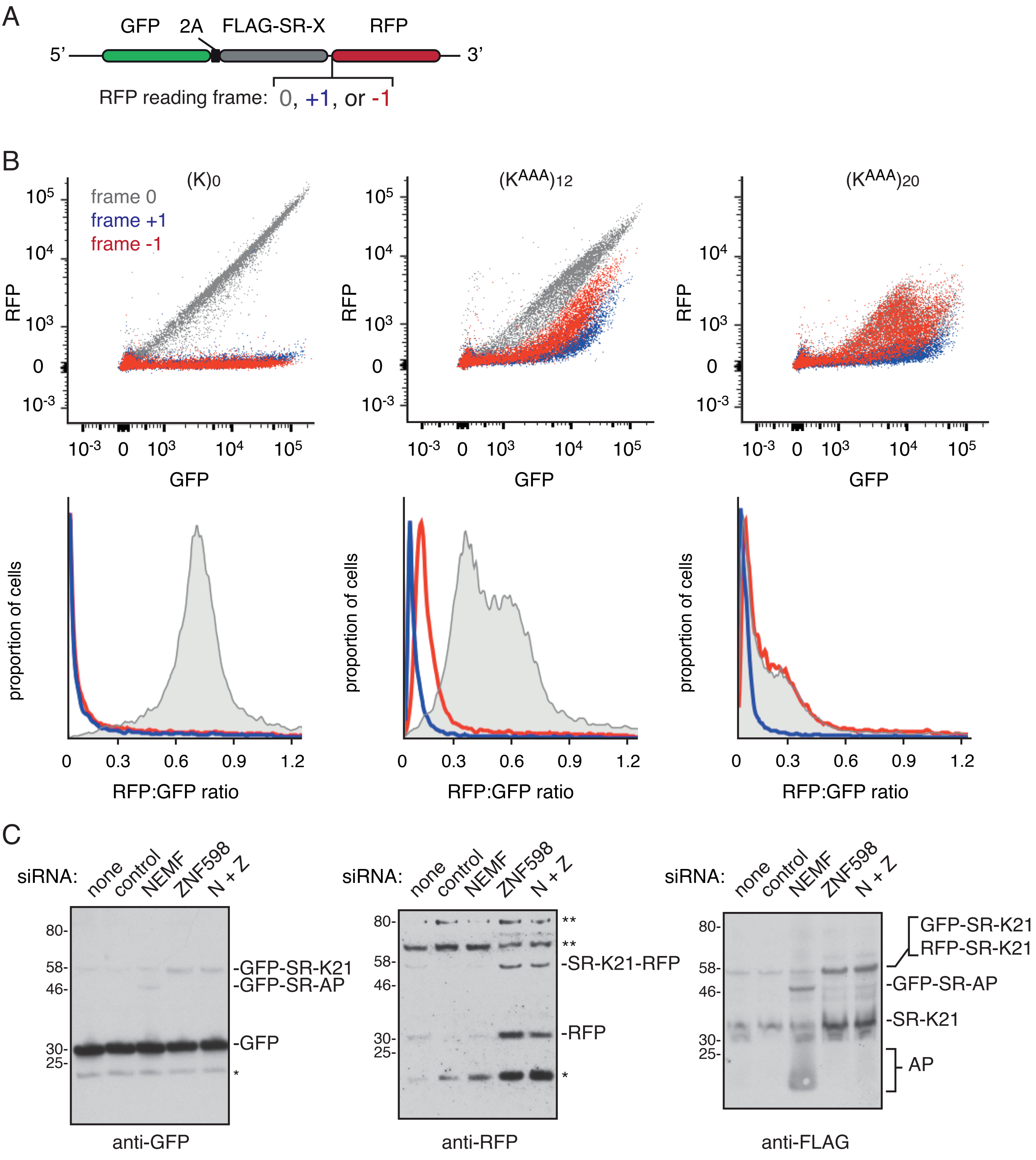
A
GFP 2A FLAG-SR-X RFP
5' 3'
RFP reading frame: 0, +1, or -1
B
(K)0
(KAAA)12
(KAAA)20
frame 0
frame +1
frame -1
RFP
GFP
proportion of cells
RFP:GFP ratio
C
siRNA: none control NEMF ZNF598 N + Z
-GFP-SR-K21
-GFP-SR-AP
-GFP
anti-GFP
-SR-K21-RFP
-RFP
anti-RFP
GFP-SR-K21
RFP-SR-K21
-GFP-SR-AP
-SR-K21
AP
anti-FLAG

Fig. S1. Characterization of the stalling reporter (related to Fig. 1).
(**A**) Schematic representation of constructs used for monitoring frameshifting. Single nucleotide insertion or deletion was introduced just preceding RFP to change its reading frame relative to the remainder of the construct. (**B**) HEK293 cells were transfected with either $(K)_0$, $(K^{AAA})_{12}$ or $(K^{AAA})_{20}$ constructs in each of the three RFP reading frames and analysed by flow cytometry after 24 hours. Shown below each scatter plot of GFP and RFP levels are the corresponding histograms of the RFP:GFP ratio. Grey is the native frame where RFP is in frame with GFP. Red and blue are constructs with RFP in the -1 and +1 frames, respectively. Note that with $(K)_0$, RFP signal is only seen when it is in the native frame. By contrast, RFP signal is detectable in each reading frame for the $(K^{AAA})_{12}$ and $(K^{AAA})_{20}$ constructs, with a slightly lower preference for the +1 frame. The RFP:GFP ratio observed at the peak of each histogram was used as an indicator of read-through into that frame. The sum of the values for all reading frames was used to estimate the net read-through (i.e., the RFP:GFP ratio that would have been seen if there was no frameshifting). This analysis shows that the compensated RFP:GFP ratio for $(K^{AAA})_{12}$ is ~88% of that seen for $(K)_0$, indicating modest stalling of ribosomes. The compensated ratio for $(K^{AAA})_{20}$ is ~43% of that seen for $(K)_0$, indicating that most ribosomes stall in this case. (**C**) The full blots for the indicated proteins are shown for cell lysates from a stable cell line expressing $(K^{AAA})_{21}$. The experiment shown is from Fig. 2B. Here, $(K^{AAA})_{21}$ cells were subjected to the indicated siRNA treatment for 72h, reporter expression induced for 24h, and analyzed by immunoblotting. If the P2A peptides work perfectly, GFP, FLAG-SR-K21, and RFP would always be separate protein products. Slight inefficiency leads to fusions of GFP with SR (detectable on both the GFP and FLAG blots), or SR with RFP (detectable on both the FLAG and RFP blots). The triple fusion was not detectable, presumably because the rate of failure at both P2A peptides is exceedingly infrequent. Double-asterisks indicate non-specific bands detected by the anti-RFP antibody. Single asterisks indicate truncated N-terminal fragments of GFP and RFP generated by unknown means, but visible in cells even expressing only these individual proteins. Note that the fusion products behave as expected in cells knocked down for either NEMF, ZNF598, or both. Thus, NEMF knockdown produces the GFP-SR arrest product (AP) visible on the FLAG and GFP blots, while ZNF598 knockdown results in increased GFP-SR-K21 and SR-K21-RFP.

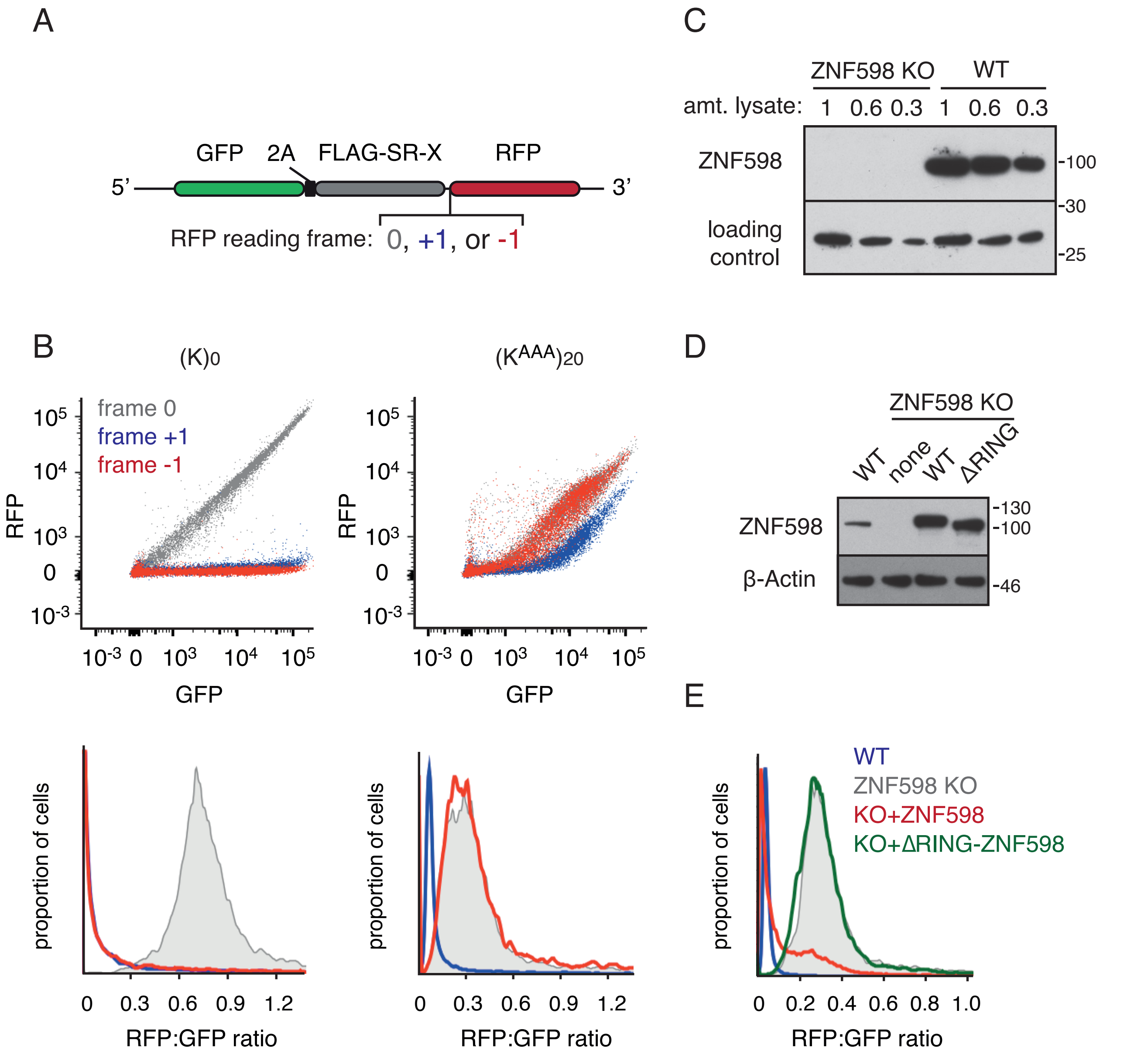

Fig. S2. Characterization of ZNF598 knockout cells (related to Fig. 2).
(**A**) Schematic representation of constructs used for monitoring frameshifting. Single nucleotide insertion or deletion was introduced just preceding RFP to change its reading frame relative to the remainder of the construct. (**B**) ZNF598 KO cells were transfected with either $(K)_0$ or $(K^{AAA})_{20}$ constructs in each of the three RFP reading frames and analysed by flow cytometry after 24h. Shown below each scatter plot of GFP and RFP levels are the corresponding histograms of the RFP:GFP ratio. Grey is the native frame where RFP is in frame with GFP. Red and blue are constructs with RFP in the -1 and +1 frames, respectively. Note that with $(K)_0$, RFP signal is only seen when it is in the native frame. By contrast, RFP signal is seen regardless of reading frame for the $(K^{AAA})_{20}$ construct, with a slightly lower preference for the +1 frame. Thus, the ribosome samples all three reading frames beyond the $(K^{AAA})_{20}$ region, indicative of frameshifting during poly(A) translation. The RFP:GFP ratio observed at the peak of each histogram was used as an indicator of read-through into that frame. The sum of the values for all reading frames was used to estimate the net read-through (i.e., the RFP:GFP ratio that would have been seen if there was no frameshifting). This analysis shows that the compensated RFP:GFP ratio for $(K^{AAA})_{20}$ is ~90% of that seen for $(K)_0$, indicating minimal stalling of ribosomes in ZNF598 knockout cells. (**C**) Serial dilutions of cell lysates from ZNF598 knockout (KO) and wild type (WT) cells were analysed by immunoblotting to confirm the complete absence of ZNF598 protein expression in the KO cells. (**D**) ZNF598 KO cells were transfected with the indicated constructs and analysed by immunoblotting after 24h to verify comparable expression levels of the wild type and ΔRING mutant ZNF598. These samples are from the experiment shown in Fig. 2D. (**E**) Histograms of the RFP:GFP ratio are plotted for the experiment in Fig. 2D relative to wild type cells (blue) analyzed in parallel. The result shows that rescue of the knockout cells with ZNF598 (red) restores the RFP:GFP ratio to wild type levels for the vast majority of cells. By contrast, ΔRING-ZNF598 has no detectable rescue and is identical to the ZNF598 knockout cells (grey versus green) .

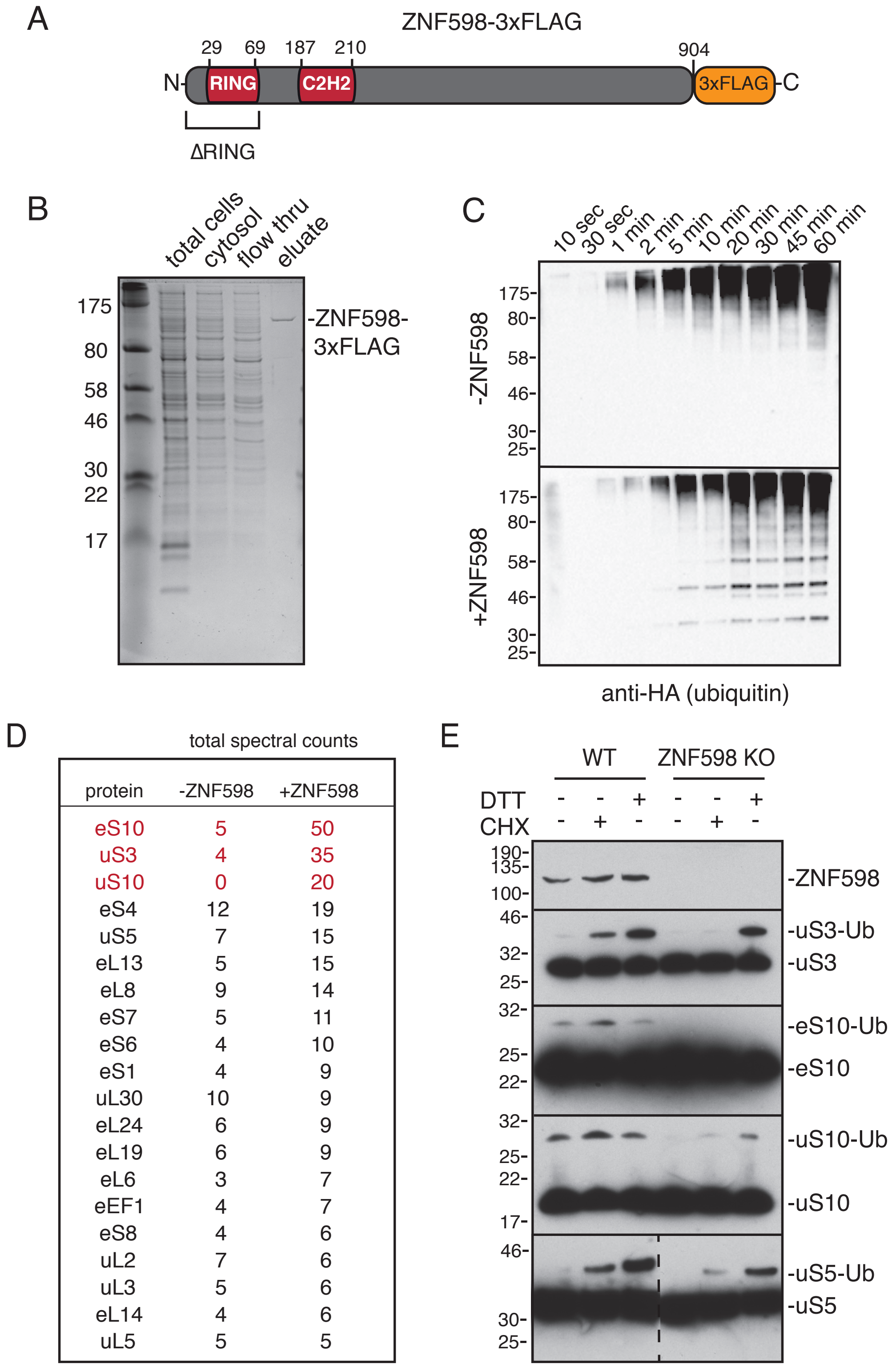

protein	-ZNF598	+ZNF598
eS10	5	50
uS3	4	35
uS10	0	20
eS4	12	19
uS5	7	15
eL13	5	15
eL8	9	14
eS7	5	11
eS6	4	10
eS1	4	9
uL30	10	9
eL24	6	9
eL19	6	9
eL6	3	7
eEF1	4	7
eS8	4	6
uL2	7	6
uL3	5	6
eL14	4	6
uL5	5	5

Fig. S3. Characterization of ribosomal protein ubiquitination (related to Fig. 3).
(**A**) Schematic representation of ZNF598 constructs used in this study illustrating the position of the affinity tag used for purification, and the region deleted to generate a ligase-inactive version. (**B**) Coomasie stained gel of fractions from a typical 3xFLAG-ZNF598 purification from transiently transfected cells. (**C**) Time course of in vitro ubiquitination of ribosomes without or with 50 nM ZNF598. The reaction included HA-ubiquitin, and the blot was probed with anti-HA. (**D**) Results of the mass spectrometry analysis of ubiquitinated proteins recovered from the experiment illustrated in Fig. 3B. Proteins identified in red showed a clear (more than five-fold) enrichment in the sample containing ZNF598. (**E**) Wild type (WT) and ZNF598 knockout (KO) cells were treated with 100 µg/ml cycloheximide or 1 mM DTT for 2 h as indicated and immunoblotted for the indicated ribosomal proteins to analyze their ubiquitination status. While uS5 was not appreciably affected in its ubiquitination under any condition by the absence of ZNF598, the other proteins were affected under some, but not all conditions. Ubiquitinated eS10 was not detected under any condition in the knockout cells.

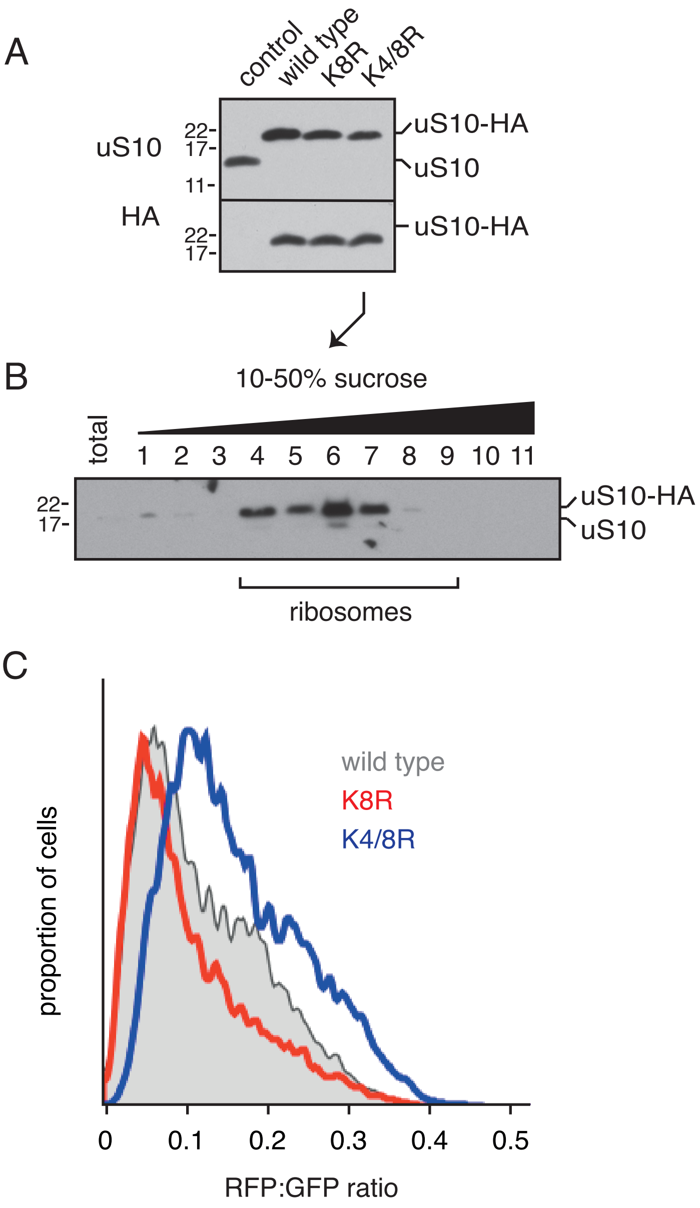

Fig. S4. Analysis of stalling in cells containing mutant uS10 (related to Fig. 4).
(**A**) Cytosol from cells stably expressing different HA-tagged variants of uS10 was analyzed by immunoblotting for uS10 and the HA tag. (**B**) Cytosol from cells stably expressing the HA-tagged K4/8R mutant of uS10 was separated on a 10-50% sucrose gradient and immunoblotted for uS10. The fractions containing ribosomes are indicated. Similar results were seen for other uS10 variants (not shown). (**C**) The indicated HA-uS10 expressing cells were transfected with the $(K^{AAA})_{20}$ reporter and analyzed by flow cytometry 24h later. The RFP:GFP ratio of all transfected cells is shown as a histogram (uS10-HA in grey, uS10-K8R-HA in red, uS10-K4/8R-HA in blue).
