# Supplementary material for: Initiation of Quality Control during Poly(A) Translation Requires Site-Specific Ribosome Ubiquitination
Source: Mol Cell. 2017 Feb 16;65(4):743–750.e4. doi: 10.1016/j.molcel.2016.11.039 (PMC5316413; doi:10.1016/j.molcel.2016.11.039)
Supplement: Document S2. Article plus Supplemental Information [file mmc2.pdf]

Short Article

Molecular Cell

Initiation of Quality Control during Poly(A) Translation Requires Site-Specific Ribosome Ubiquitination

Graphical Abstract

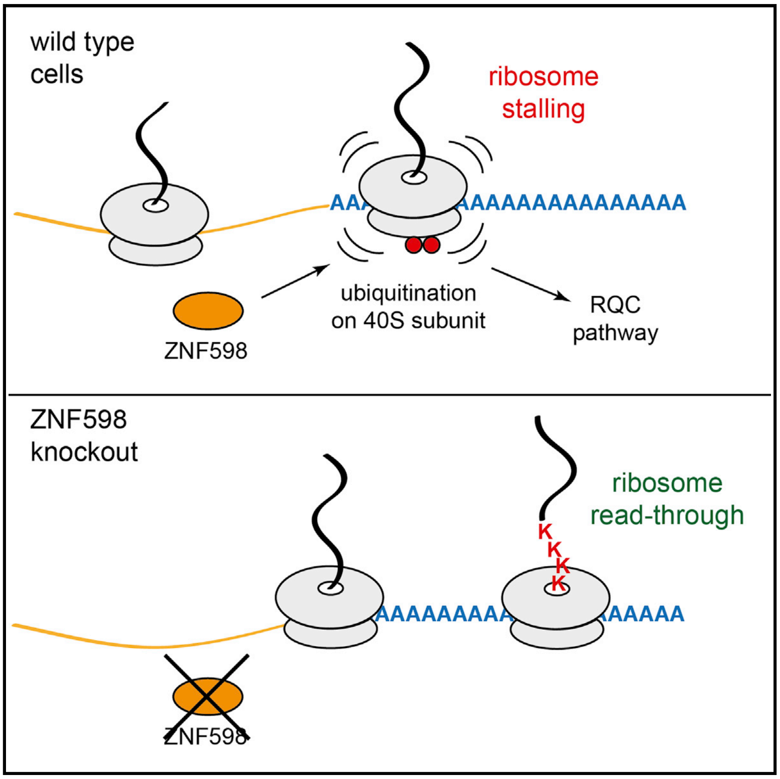

Authors

Szymon Juszkiewicz,
Ramanujan S. Hegde

Correspondence

rhegde@mrc-lmb.cam.ac.uk

In Brief

Juszkiewicz and Hegde report that ZNF598 mono-ubiquitinates ribosomal protein eS10 to stall translation on poly(A) sequences. Stalling precludes synthesis of a long poly-lysine tail, instead initiating the ribosome-associated quality control pathway to degrade the potentially detrimental aberrant nascent polypeptide.

Highlights

- Poly(A), not poly-basic tracts, are the main trigger of ribosome stalling in mammals
- The ubiquitin ligase ZNF598 is required to stall ribosomes during poly(A) translation
- ZNF598 primarily mono-ubiquitinates two lysines on the 40S ribosomal protein eS10
- ZNF598 deletion or mutation of its eS10 target permits increased poly(A) translation

Juszkiewicz & Hegde, 2017, Molecular Cell *65*, 743–750
February 16, 2017 © 2016 The Authors. Published by Elsevier Inc.
http://dx.doi.org/10.1016/j.molcel.2016.11.039

Molecular Cell
Short Article

CellPress

Initiation of Quality Control during Poly(A) Translation Requires Site-Specific Ribosome Ubiquitination

Szymon Juszkiewicz[1] and Ramanujan S. Hegde[1,2,*]
[1]MRC Laboratory of Molecular Biology, Cambridge CB2 0QH, UK
[2]Lead Contact
*Correspondence: rhegde@mrc-lmb.cam.ac.uk
http://dx.doi.org/10.1016/j.molcel.2016.11.039

SUMMARY

Diverse cellular stressors have been observed to trigger site-specific ubiquitination on several ribosomal proteins. However, the ubiquitin ligases, biochemical consequences, and physiologic pathways linked to these modifications are not known. Here, we show in mammalian cells that the ubiquitin ligase ZNF598 is required for ribosomes to terminally stall during translation of poly(A) sequences. ZNF598-mediated stalling initiated the ribosome-associated quality control (RQC) pathway for degradation of nascent truncated proteins. Biochemical ubiquitination reactions identified two sites of mono-ubiquitination on the 40S protein eS10 as the primary ribosomal target of ZNF598. Cells lacking ZNF598 activity or containing ubiquitination-resistant eS10 ribosomes failed to stall efficiently on poly(A) sequences. In the absence of stalling, read-through of poly(A) produces a poly-lysine tag, which might alter the localization and solubility of the associated protein. Thus, ribosome ubiquitination can modulate translation elongation and impacts co-translational quality control to minimize production of aberrant proteins.

INTRODUCTION

Translation of proteins is a fundamental and energetically costly process that is regulated at many levels and subject to stringent quality control. Although post-translational protein quality control has been extensively studied, quality control of nascent polypeptides during translation has only recently emerged (Brandman and Hegde, 2016; Wang et al., 2015). Co-translational protein quality control has its origins in mRNA surveillance pathways for the degradation of aberrant transcripts (Shoemaker and Green, 2012). A major cue for detection of an aberrant mRNA is the stalling of a translating ribosome. Although the mechanisms are only partially known, a stalled ribosome can initiate not only degradation of the associated mRNA (Doma and Parker, 2006) but also recycling of the ribosomal subunits (Pisareva et al., 2011; Shoemaker et al., 2010) and degradation of the partially synthesized nascent polypeptide (Bengtson and Joazeiro, 2010; Ito-Harashima et al., 2007).

Several genetic, biochemical, and structural studies have begun to identify the main factors, steps, and mechanisms downstream of a stalled ribosome culminating in nascent protein degradation (reviewed in Brandman and Hegde, 2016). A key event in providing the ubiquitination machinery access to the nascent protein is separation of the ribosomal subunits (Shao et al., 2013). This reaction can be mediated by the ribosome rescue factors Pelota (Dom34 in yeast), Hbs1, and ABCE1 (Rli1 in yeast) (Pisareva et al., 2011; Shoemaker and Green, 2011; Shoemaker et al., 2010), although other rescue pathways probably exist (Chiabudini et al., 2014; Shcherbik et al., 2016). Once the 40S is removed, the intersubunit surface of the 60S-nascent chain complex is recognized by NEMF (Rqc2 in yeast), whose interaction with the ubiquitin ligase Ltn1 facilitates its stable 60S binding (Lyumkis et al., 2014; Shao et al., 2015; Shen et al., 2015). Ltn1 then ubiquitinates the nascent protein (Bengtson and Joazeiro, 2010; Brandman et al., 2012; Shao and Hegde, 2014), whose eventual extraction and proteasome degradation requires the AAA-ATPase p97 (Cdc48 in yeast) and Rqc1 (Defenouillère et al., 2013; Verma et al., 2013).

Thus, the key trigger of the ribosome-associated quality control (RQC) pathway is a terminally stalled ribosome acted on by ribosome rescue factors. For some mRNAs defects, such as truncation within the coding region, the mechanism of stalling is a simple physical impediment. In other cases, however, the determinants for terminal stalling, and hence initiation of the RQC pathway, are more nuanced. One example is stalling at poly(A) sequences, which might be needed to initiate RQC in instances of inappropriate poly-adenylation at cryptic sites within an open reading frame. Early studies in yeast suggested that stalling was due to the translation of poly-basic residues (AAA codes for lysine) independent of codon choice (Ito-Harashima et al., 2007). The mechanism was proposed to involve electrostatic interactions between a poly-basic nascent chain and the RNA-lined exit tunnel in the ribosome (Lu and Deutsch, 2008).

Although such a mechanism might play a contributory role, several recent findings indicate a more complex mechanism of stalling. First, synonymous codon choice can strongly impact

CrossMark

© 2016 The Authors. Published by Elsevier Inc.
This is an open access article under the CC BY license (http://creativecommons.org/licenses/by/4.0/).

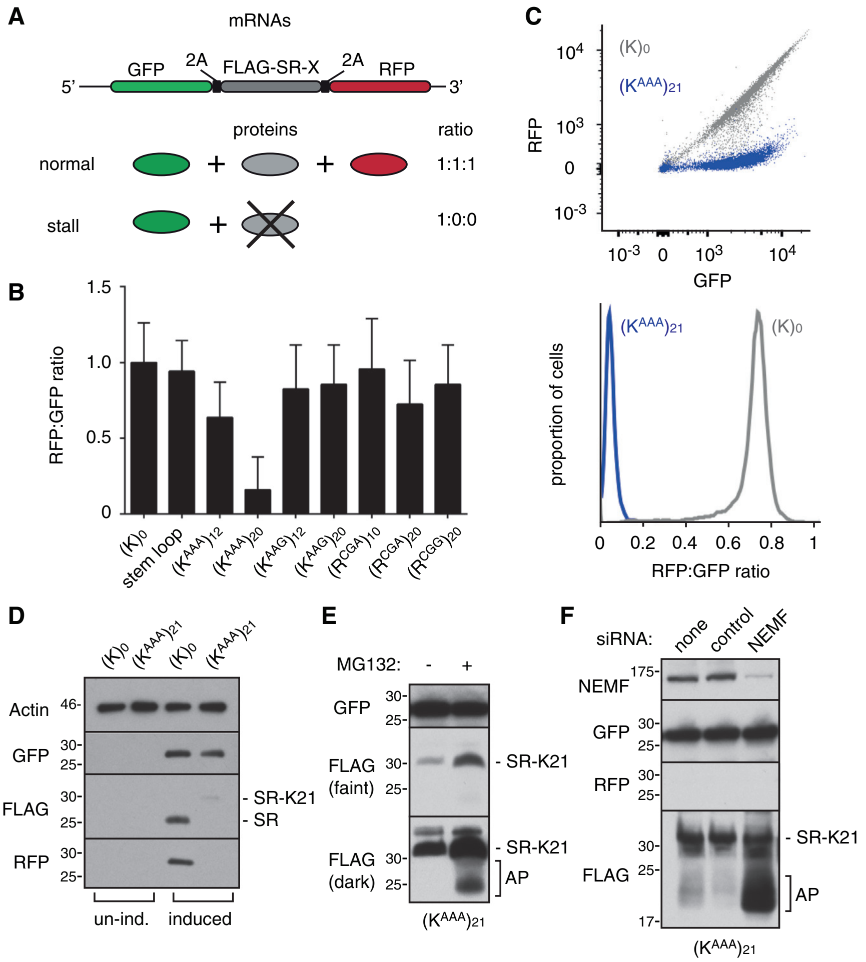

Figure 1. Assay of Terminal Ribosome Stalling at Single-Cell Resolution

(A) Diagram of the reporter construct and expected protein products in the absence or presence of terminal stalling.

(B) Median RFP:GFP ratio of 20,000 transfected cells transiently expressing the reporter construct containing the indicated test sequences. Error bars represent 68% of the events around the median.

(C) Isogenic stable cell lines expressing the $(K)_0$ (in gray) or $(K^{AAA})_{21}$ (in blue) reporter for 24 hr were analyzed by flow cytometry. A scatterplot of individual cells (top) and a histogram of GFP:RFP ratio (bottom) are shown. This and all other scatterplots are shown on a bi-exponential scale to better visualize data across the wide range of expression levels seen in these experiments.

(D) Immunoblot of $(K)_0$ and $(K^{AAA})_{21}$ cells without and with induction of reporter expression with doxycycline for 24 hr.

(E) $(K^{AAA})_{21}$ expressing cells were treated with 20 μM MG132 for 4 hr and analyzed by immunoblotting for the indicated products.

(F) $(K^{AAA})_{21}$ cells were subjected to small interfering RNA (siRNA) treatment against the indicated targets for 72 hr, reporter expression induced for 24 hr, and analyzed by immunoblotting for the indicated proteins. See also Figure S1.

stalling (Arthur et al., 2015; Letzring et al., 2013). For example, the CGA arginine codon is particularly potent in yeast (Letzring et al., 2013), whereas AAA lysine codons are more effective than AAG in mammalian cells (Arthur et al., 2015). Second, deletion of the ribosomal protein Asc1 or the ubiquitin ligase Hel2 permits read-through of poly-basic sequences (Brandman et al., 2012; Kuroha et al., 2010; Letzring et al., 2013). Third, the position of a poly-basic sequence within an open reading frame influences the dependence of stalling on Asc1/Hel2 (Letzring et al., 2013). These observations are difficult to reconcile with a physical obstruction model of poly-basic stalling and suggest a role for other factor(s).

In this context, the recent observation that ribosomes can be ubiquitinated at specific sites under different stress conditions is especially noteworthy (Higgins et al., 2015). Some of the stressors (such as translation elongation inhibition) impact translation directly, while others (such as proteasome or endoplasmic reticulum [ER] stress) do so indirectly. This led to the speculation that site-specific ubiquitination on ribosomal proteins might influence, or be influenced by, translation. Given that stalling of ribosomes in yeast is influenced by Hel2 (Brandman et al., 2012; Letzring et al., 2013), a ubiquitin ligase of unknown function or specificity, we considered the idea that translation elongation and ribosome ubiquitination might be mechanistically linked in the RQC pathway. Here, we show that ZNF598, a ligase with partial homology to Hel2, is required for stalling at poly(A) sequences. By identifying the ribosomal sites for ZNF598, we could causally link site-specific mono-ubiquitin on the 40S subunit to modulation of translation elongation at an early step of the RQC pathway.

RESULTS

Poly(A) Is the Major Trigger for Initiation of Mammalian RQC

We developed a flow cytometry-based assay to quantitatively assay terminal ribosome stalling at single-cell resolution in mammalian cells (Figure 1A). Our reporter cassette contains N- and C-terminal GFP and RFP markers separated by a FLAG-tagged stalling reporter (SR) that has been characterized in cell free systems (Shao et al., 2015). FLAG-SR is insulated by viral P2A sequences, at which ribosomes skip formation of a peptide bond without interrupting translation elongation (Lin et al., 2013). Thus, complete translation of the cassette will generate three proteins (GFP, FLAG-SR, and RFP) in equal amounts. By contrast, terminal stalling during FLAG-SR translation would abort translation prior to RFP synthesis, resulting in a sub-stoichiometric RFP:GFP ratio. Because GFP and RFP have long and comparable half-lives and are not attached to potential degrons, their ratio is expected to be a faithful reflection of terminal (i.e., abortive) stalling at the intervening sequence. Translation of GFP, which would be complete and released prior to

encountering FLAG-SR, would indirectly reflect mRNA abundance. Finally, the levels of FLAG-SR normalized to GFP can report on its relative degradation by cellular quality control pathways.

In initial experiments to identify suitable stalling sequences, we tested several constructs by flow cytometry in transiently transfected HEK293T cells. Two different arginine codons (CGG and CGA) were impervious to stalling at repeat lengths up to 20, and a stable stem-loop that induces strong stalling in yeast (Doma and Parker, 2006) was ineffective in mammals. Only strings of AAA lysine codons [denoted $(K^{AAA})_n$, where n is the number of codons] caused an appreciable decrease in the RFP:GFP ratio relative to the reporter lacking an insert (Figure 1B). $(K^{AAA})_{12}$ induced only modest decrease in the RFP:GFP ratio, $(K^{AAA})_{20}$ showed ~8-fold decrease, whereas $(K^{AAG})_{20}$ was essentially inert. Because of possible frameshifting at sequential AAA codons (Arthur et al., 2015; Koutmou et al., 2015), the RFP:GFP ratio is likely to underestimate the overall level of read-through. When correcting for this effect by quantifying RFP production in each reading frame, we can deduce that $(K^{AAA})_{12}$ and $(K^{AAA})_{20}$ show ~10% and ~60% stalling, respectively (Figures S1A and S1B).

These findings are generally consistent with recent findings using somewhat different reporters showing that AAA is the most potent trigger of stalling in mammalian cells (Arthur et al., 2015). Whether the lower threshold for stalling (as few as six AAA codons in this earlier work) is due to a different reporter system, different positioning (near the 5′ end of the coding region), or different cell types remains to be clarified. Nevertheless, we can conclude from the available data that unlike in yeast, stalling within an mRNA is strongly tuned to favor poly(A). In our experiments, the threshold for stalling is clearly higher than any poly(A) regions encoded within the mammalian genome. This suggests that mRNAs that have been promiscuously poly-adenylated in the coding region would probably trigger stalling before synthesizing ~20 lysines.

To investigate poly(A)-mediated stalling in greater detail, we prepared matched stable doxycycline-inducible cell lines in which the reporter lacking or containing 21 AAA lysine codons (termed $(K)_0$ and $(K^{AAA})_{21}$, respectively) was inserted into the same integration site of HEK293 cells. Flow cytometry showed the expected correlation between GFP and RFP levels across a wide expression range for $(K)_0$ but strongly reduced RFP for $(K^{AAA})_{21}$ (Figure 1C). Immunoblotting verified the flow cytometry results and further showed that FLAG-SR was notably lower in $(K^{AAA})_{21}$ cells relative to $(K)_0$ (Figure 1D). The small amount seen in $(K^{AAA})_{21}$ corresponds to the size expected for read-through of the poly-basic region (to generate FLAG-SR-K_{21}), which appears to occur at a low level consistent with the low level of RFP detectable by flow cytometry. Inspection of higher molecular weight regions of such blots (Figure S1C) showed that peptide bond skipping at the P2A sequences failed at a detectable level, resulting in fused rather than separated proteins (Lin et al., 2013). This was minor and does not interfere appreciably with the flow cytometry assay or FLAG-SR fate.

Proteasome inhibition resulted in a small increase of the full length FLAG-SR-K_{21} read-through product and the appearance of a heterogeneous set of lower molecular weight products (hereafter termed arrest products [AP]) (Figure 1E). Only the arrest products were stabilized in cells knocked down for NEMF (Figure 1F), an essential component of RQC-mediated nascent chain degradation. These observations indicate that most ribosomes stall when they encounter the $(K^{AAA})_{21}$ sequence and that the arrest products on these stalled ribosomes are degraded by the RQC pathway. Of the small amount that reads through, the resulting SR-K_{21} product appears to be targeted for proteasomal degradation via the K_{21} tag acting as a degron, highlighting the importance of insulating the fluorescent reporters from the test sequence for monitoring stalling. Of note, the GFP levels are essentially unchanged in $(K)_0$ versus $(K^{AAA})_{21}$ cells (Figures 1C and 1D), suggesting that at least in HEK293 cells, engagement of the RQC pathway is not accompanied by appreciable mRNA destabilization. The basis for this uncoupling of mRNA and protein quality control is not clear at this time but argues that the stalling-dependent endonucleolytic cleavage step thought to trigger mRNA decay (Doma and Parker, 2006; Kuroha et al., 2010; Tsuboi et al., 2012) is not a prerequisite for initiation of the RQC pathway.

ZNF598 Facilitates Stalling on Poly(A) Sequences

With a quantitative assay and validated substrate, we investigated the potential role of ubiquitination in poly(A)-mediated ribosome stalling. In yeast, the ubiquitin ligase Hel2 has been shown to influence read-through of CGA codons (Brandman et al., 2012; Letzring et al., 2013). Although standard BLAST searches did not reveal a clear mammalian homolog of Hel2, the Ensembl Biomart tool (Flicek et al., 2014) identified the human RING domain containing protein ZNF598 as a potential candidate (~15% identity).

Knockdown of ZNF598 in the stable cell line expressing the $(K^{AAA})_{21}$ construct showed an increased RFP:GFP ratio (Figure 2A) and increased levels of full-length FLAG-SR-K_{21} (Figure 2B), indicating read-through of the poly(A) region. After compensating the RFP signal for frameshifting that occurs during poly(A) translation (Figures S2A and S2B), we estimate that read-through of $(K^{AAA})_{21}$ in the absence of ZNF598 is ~90% of that seen for the $(K)_0$ construct. Thus, almost no terminal stalling at $(K^{AAA})_{21}$ occurs in ZNF598 knockdown cells.

Combined knockdown of NEMF and ZNF598 led to the disappearance of the arrest fragments seen with NEMF knockdown alone (Figure 2B). This implies that ZNF598 acts upstream of and initiates the RQC pathway. Using ZNF598 knockout cells (Figure S2C) that recapitulate the knockdown phenotype (Figure 2C), we could demonstrate near-complete rescue by re-expressing wild-type ZNF598 but not a ligase-deficient mutant expressed at comparable levels (Figure 2D; Figures S2D and S2E). Thus, the ability of ribosomes to stall efficiently at poly(A) and initiate the RQC pathway appears to be almost completely dependent on the ubiquitin ligase activity of ZNF598.

eS10 Is the Primary Ribosomal Target of ZNF598 In Vitro and In Vivo

We next investigated the potential target(s) of ZNF598 that might modulate translation arrest at poly(A) sequences. We found that purified human ZNF598 (Figures S3A and S3B) was capable of adding tagged ubiquitin to purified mammalian ribosomes

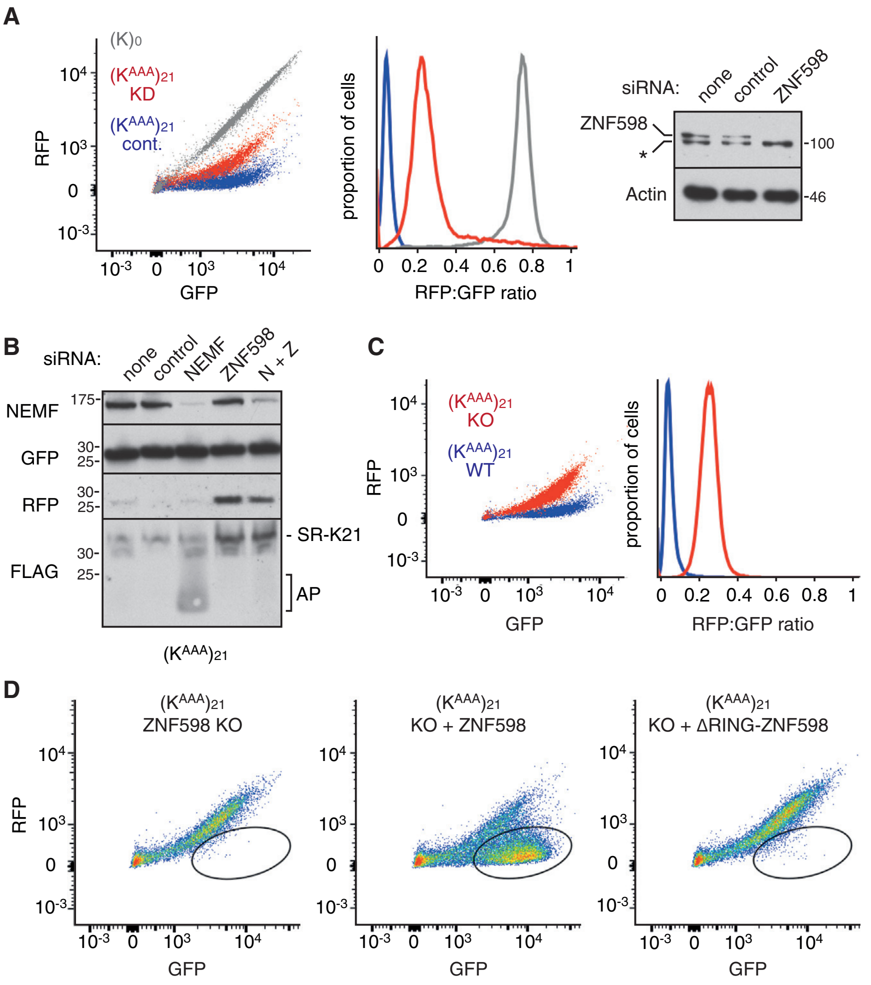

Figure 2. ZNF598 Is Required to Initiate the RQC Pathway during Poly(A) Translation

(A) $(K^{AAA})_{21}$ cells were transfected with control (blue) or ZNF598 (red) siRNAs for 72 hr, reporter expression induced for 24 hr, and analyzed by flow cytometry (left and middle panels) or immunoblotting for the indicated proteins (right panel). $(K)_0$ cells (gray) served as a reference.

(B) $(K^{AAA})21$ cells were subjected to the indicated siRNA treatment for 72 hr, reporter expression induced for 24 hr, and analyzed by immunoblotting for the indicated proteins.

(C) $(K^{AAA})_{21}$ reporter expression was induced for 24 hr in wild-type (WT, in blue) and ZNF598 knockout (KO, in red) cells and analyzed by flow cytometry.

(D) $(K^{AAA})_{21}$ cells knocked out for ZNF598 were transiently transfected with the indicated plasmids and the transfected cells (as judged by a co-transfected BFP construct) were analyzed by flow cytometry after reporter induction for 24 hr. The majority of cells displayed restoration of terminal ribosomal stalling to wild-type levels (circled). See also Figure S2.

in vitro (Figure 3A; Figure S3C). In addition to the heterogeneous mixture of ubiquitin conjugates apparently added by endogenous ribosome-associated ligases, two prominent ZNF598-dependent ubiquitinated products were observed (Figure 3A, left panel). Both ubiquitinated proteins and ZNF598 were observed in ribosomal fractions (Figure 3A, right panel).

Purification of ubiquitinated proteins from the ribosomal fractions under denaturing conditions via the tagged ubiquitin revealed two major bands that collapsed to a single product of ~20 kDa upon treatment with a promiscuous de-ubiquitinase (Figure 3B). This product was identified by mass spectrometry to be eS10 (also called RPS10) and was verified by di-Gly-modified residues to have been ubiquitinated on K138 and/or K139. Although not visualized prominently on the stained gel, mass spectrometry of the purified ZNF598-stimulated ubiquitin conjugates (Figure S3D) also identified uS10 (RPS20; modified on K4 and K8) and uS3 (RPS3; modified on K214). Immunoblotting of the in vitro ubiquitination reaction products verified ZNF598-mediated ubiquitination of eS10 and uS10 with mono-ubiquitin at up to two sites, whereas other ribosomal proteins were unaffected (Figure 3C). Appreciable uS3 ubiquitination by ZNF598 was not seen by immunoblotting (data not shown).

Immunoblotting of eS10, uS10, uS3, and uS5 (also called RPS2) in HEK293 cells revealed minor ~10 kDa larger products for each that were diminished or absent in cells lacking ZNF598 (except uS5 which was not affected by ZNF598 absence) (Figure 3D; Figure S3E). These products were verified to be ubiquitin by the appearance of a slightly slower migrating species in cells transfected with hemagglutinin (HA)-tagged ubiquitin, and the recovery of HA-ubiquitinated versions of eS10 (Figure 3E), uS3, and uS10 (data not shown). Treatment of cells with the elongation inhibitor cycloheximide or the ER stressor DTT, both shown earlier to stimulate ribosome ubiquitination (Higgins et al., 2015), affected each protein in somewhat different ways. The modest increase in ubiquitination of each protein with cycloheximide was attenuated (uS3 and uS10) or entirely abolished (eS10) in ZNF598 knockout cells. The ubiquitination levels of uS3 and uS10 in DTT-treated cells was ZNF598 independent. Thus, there are multiple (spatially nearby) targets for ZNF598, yet unidentified ligases for the ribosome and stress-dependent and -independent sites of modification. Of these, eS10 is the most efficient ZNF598 target in vitro and the most ZNF598-dependent in vivo.

Efficient Poly(A)-Mediated Stalling Requires eS10 Ubiquitination

We generated matched isogenic stable cell lines expressing HA-tagged wild-type eS10 or versions containing lysine to arginine mutants at the identified ZNF598 target sites. At steady state, exogenous eS10 comprises ~70% of total eS10 (whose overall levels remain constant) and is incorporated efficiently into the ribosomes (Figures 4A and 4B). Analysis of the $(K^{AAA})_{21}$ reporter

CellPress

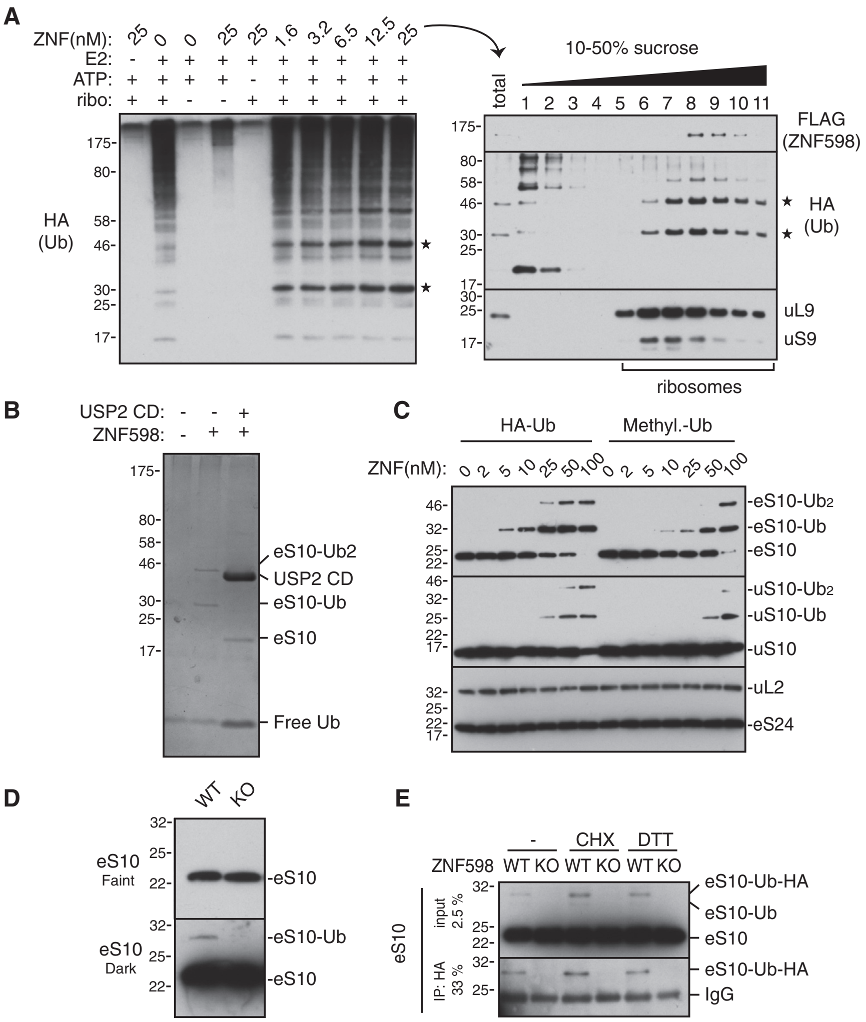

Figure 3. ZNF598 Ubiquitinates eS10 In Vitro and In Vivo

(A) In vitro ubiquitination reactions of ribosomes using HA-tagged ubiquitin and the indicated factors were analyzed by immunoblotting with anti-HA to detect all new ubiquitin conjugates. The last reaction was also separated on a 10%–50% sucrose gradient and analyzed by immunoblotting for the indicated antigens (right panel). Asterisks indicate primary ribosomal ubiquitin conjugates.

(B) Ribosome ubiquitination reactions with His-tagged ubiquitin in the presence (+) or absence (−) of ZNF598 were fractionated to purify ubiquitinated core ribosomal proteins, treated or untreated with the catalytic domain (CD) of the deubiquitinase Usp2, and analyzed by SDS-PAGE and Coomassie blue staining. The major ZNF598-dependent products were identified by mass spectrometry.

(C) Ribosomes were ubiquitinated with increasing concentrations of ZNF598 with either HA-tagged ubiquitin or methyl-ubiquitin, and analyzed by immunoblotting for the indicated ribosomal proteins.

(D) eS10 ubiquitination status in WT and ZNF598 KO cells was analyzed by immunoblotting. Two exposures are shown.

(E) WT or ZNF598 KO cells were transfected with HA-ubiquitin and treated with nothing, 100 μg/ml cycloheximide, or 1 mM DTT for 2 hr. Anti-eS10 immunoblots are shown for total cell lysate and anti-HA affinity purified products. The positions of ubiquitin- or HA-ubiquitin-modified eS10 are indicated. See also Figure S3.

showed increased RFP:GFP ratio in the eS10-(K139R) cells and a further increase in the K138R/K139R double mutant (Figure 4C). Given that ~30% of ribosomes still contain endogenous eS10, and poly(A)-mediated frameshifting underestimates read-through, the seemingly modest effect on the RFP:GFP ratio in the ubiquitination-resistant eS10 cells actually represents substantial read-through. Nevertheless, the effect is not as complete as ZNF598 knockout, suggesting that other ubiquitination sites might partially compensate. Consistent with this idea, we can observe a partial read-through in K4R/K8R double-mutant uS10 cells (Figures S4A–C). We conclude that mutation of the most efficient ubiquitination targets on the ribosome by ZNF598 partially phenocopies ZNF598 deletion with respect to translation of poly(A) sequences.

DISCUSSION

We have identified ZNF598 as a ubiquitin ligase for the mammalian ribosome, defined eS10 as a primary target for mono-ubiquitination, and demonstrated a requirement for this modification in ribosome stalling at poly(A) sequences. These findings provide direct evidence that translation elongation is subject to regulation by *trans*-acting factors that operate on a core ribosomal protein. This conclusion has a number of implications for both general translation and nascent protein quality control and opens up several important directions for future study.

We find that the main sequence feature that induces terminal ribosome stalling is poly(A). Although this had long been speculated to be a consequence of a poly-basic nascent chain interacting with the negatively charged ribosomal exit tunnel (Lu and Deutsch, 2008), this model would seem overly simplistic, at least in mammalian cells. Quantitative single-cell analysis of terminal ribosome stalling in HEK293 cells unambiguously shows that neither poly-lysine nor poly-arginine, at lengths beyond any found in the genome, stall translation appreciably. Although it remains possible that poly-basic stretches slow translation, this is apparently insufficient to trigger RQC in HEK293 cells. Instead, only translation of poly(A) sequences longer than ~40 nt are effective triggers of RQC. This length cutoff is notable because there are no native sequences of this length, whereas essentially all poly(A) tails are longer. Thus, the mechanism of stall detection appears to be tuned to detect translation of the poly(A) tail with high specificity.

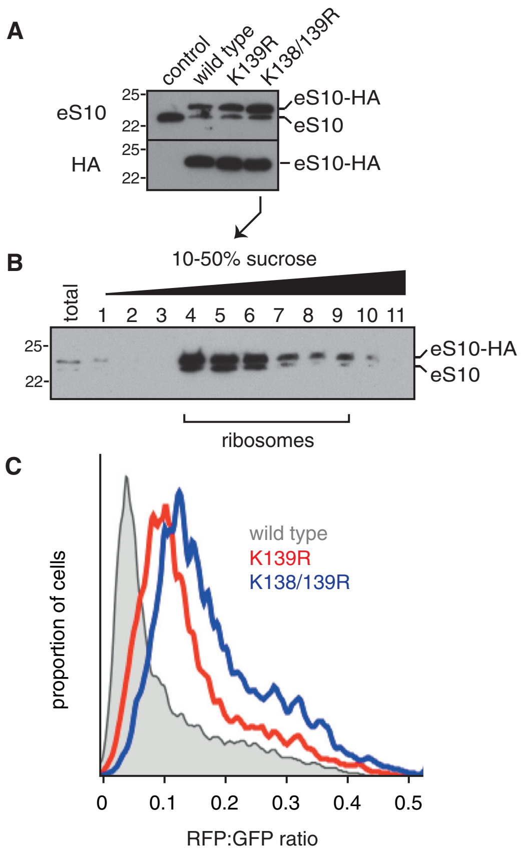

Figure 4. Ubiquitination of eS10 Facilitates Initiation of the RQC Pathway

(A) Cytosol from cells stably expressing different HA-tagged variants of eS10 was analyzed by immunoblotting for eS10 and the HA tag.

(B) Cytosol from cells stably expressing the HA-tagged K138/139R mutant of eS10 was separated on a 10%–50% sucrose gradient and immunoblotted for eS10. The fractions containing ribosomes are indicated. Similar results were seen for other eS10 variants (not shown).

(C) The indicated HA-eS10 expressing cells were transfected with the $(K^{AAA})_{20}$ reporter and analyzed by flow cytometry 24 hr later. The RFP:GFP ratio of all transfected cells is shown as a histogram (eS10-HA in gray, eS10-K139R-HA in red, eS10-K138/139R-HA in blue). See also Figure S4.

It is not entirely clear why it is important for the cell to stall at poly(A) tails given that it would eventually stall at the 3′ end and engage the RQC pathway. Indeed, mRNAs lacking a stop codon (non-stop decay [NSD] substrates) do not depend on Hel2 to engage the RQC pathway in yeast (Saito et al., 2015; Yonashiro et al., 2016). Whether RQC was engaged via stalling within or at the end of the poly(A) tail was not determined. In either case, however, the poly-lysine sequence would be relatively short and contained within the ribosomal tunnel given that poly(A) tails in yeast are short (median length of 27 nt) and never exceed ~90 nt (Subtelny et al., 2014). Thus, nascent proteins of NSD substrates in yeast would be degraded without or with stalling before poly-lysine is exposed to the cytosol.

In mammals, however, newly made mRNAs contain a poly(A) tail of ~250 nt. This corresponds to over 80 lysines, more than half of which would emerge from the ribosome if stalling did not occur on NSD substrates. Because poly-lysine can be a potent nuclear/nucleolar targeting signal, stalled ribosomes exposing such sequences may inappropriately engage nuclear import factors, a fate that can be avoided by stalling at a point when the poly-lysine is still within the ribosomal exit tunnel. We therefore speculate that synthesis of extended poly-lysine might be detrimental and under negative selection. Indeed, basic poly-dipeptides (glycine-arginine or proline-arginine) encoded by the neurodegeneration-associated expansion repeats of the *C9orf72* gene accumulate in nucleolar puncta and impede RNA biogenesis (Kwon et al., 2014; Mizielinska et al., 2014), perhaps providing another rationale for limiting poly(A) translation. This could be especially important in mammals, where complex mRNA splicing and multiple cryptic polyadenylation sites might lead to frequent premature polyadenylation (Kaida et al., 2010).

Given that mutation of the primary (eS10) or secondary (uS10) lysines modified by ZNF598 permits read-through, we conclude that ribosome ubiquitination of at least one of these sites is needed for terminal stalling at poly(A). Although we cannot entirely exclude a non-ubiquitin role of these lysine residues that is lost upon mutation to arginine, the similar phenotypes in both ZNF598 knockout cells and ribosomal mutant cells argues strongly for ubiquitin as the key trigger of stalling. The fact that one can see effects with either eS10 or uS10 mutants, together with the modified sites residing on flexible tails of these proteins, further suggests that the precise position of the ubiquitin is not critical. This would suggest a model where ubiquitin in this region of the solvent side of the 40S ultimately inhibits elongation. Because the key functional regions for elongation (the mRNA channel, and A, P, and E sites) are relatively distant, the mechanism of stalling is unlikely to be simple obstruction of a biochemical reaction. Instead, it seems plausible that other factors are needed to communicate the ubiquitination status of these sites to the translation machinery. The quantitative fluorescent assay described here should facilitate mammalian genetic screens for additional machinery to eventually deduce the mechanism of ubiquitin-directed stalling.

In a broader sense, the discovery that a site-specific ubiquitin on the ribosome can impact elongation raises the possibility of translational regulation for non-quality control reasons. Although our current analysis has directly linked eS10 (and to a lesser extent, uS10) ubiquitination to poly(A) stalling, it is plausible that elongation through other sequences are also sensitive to ubiquitination status. Indeed, a recently emerging area is that codon optimality can influence mRNA stability by yet unknown mechanisms that depend on its translation (Presnyak et al., 2015). It is therefore attractive to postulate that a combination of codon optimality and ligase activity is employed to regulate elongation in an mRNA-selective manner to control protein and/or mRNA levels. Transcriptome-wide ribosome profiling in ZNF598 null cells may shed light on such regulation.

Finally, it is important to note that only certain ribosomal ubiquitination sites were affected by ZNF598 deletion. Such ZNF598-independent sites include both constitutive and stress-responsive ubiquitination. It will be of considerable interest to link each of these sites to a ribosome-associating ubiquitin ligase and physiologic outcome as we have done with ZNF598 and

poly(A) stalling. Collectively, these and other types of ribosome modifications appear to represent an underappreciated means of translational regulation for future study.

STAR★METHODS

Detailed methods are provided in the online version of this paper and include the following:

- KEY RESOURCES TABLE
- CONTACT FOR REAGENT AND RESOURCE SHARING
- EXPERIMENTAL MODEL AND SUBJECT DETAILS
  - Cell Lines
- METHOD DETAILS
  - Constructs
  - Cell Cultures
  - Flow Cytometry Analysis
  - Western Blot Analysis
  - Ribosomes Purification
  - Recombinant ZNF598-3X-FLAG Protein Purification
  - In Vitro Ubiquitination of Ribosomes
  - Sucrose Gradient Fractionation
  - Cytosol Fractionation
- QUANTIFICATION AND STATISTICAL ANALYSIS

SUPPLEMENTAL INFORMATION

Supplemental Information includes four figures and can be found with this article online at http://dx.doi.org/10.1016/j.molcel.2016.11.039.

AUTHOR CONTRIBUTIONS

S.J. designed and conducted the experiments; S.J. and R.S.H. conceived the project, interpreted the results, and wrote the manuscript.

ACKNOWLEDGMENTS

We thank V. Bittl for constructs and initial assay development, M. Daly and E. Zavodszky for help with flow cytometry, M. Skehel for mass spectrometry, S. Shao for ubiquitination advice, K. Yanagitani for constructs, and Hegde lab members for useful discussions. This work was supported by UK Medical Research Council Grant MC_UP_A022_1007 (to R.S.H.).

Received: September 25, 2016
Revised: November 16, 2016
Accepted: November 28, 2016
Published: January 5, 2017

REFERENCES

Arthur, L., Pavlovic-Djuranovic, S., Smith-Koutmou, K., Green, R., Szczesny, P., and Djuranovic, S. (2015). Translational control by lysine-encoding A-rich sequences. Sci. Adv. *1*, e1500154.

Bengtson, M.H., and Joazeiro, C.A. (2010). Role of a ribosome-associated E3 ubiquitin ligase in protein quality control. Nature *467*, 470–473.

Brandman, O., and Hegde, R.S. (2016). Ribosome-associated protein quality control. Nat. Struct. Mol. Biol. *23*, 7–15.

Brandman, O., Stewart-Ornstein, J., Wong, D., Larson, A., Williams, C.C., Li, G.-W., Zhou, S., King, D., Shen, P.S., Weibezahn, J., et al. (2012). A ribosome-bound quality control complex triggers degradation of nascent peptides and signals translation stress. Cell *151*, 1042–1054.

Chakrabarti, O., and Hegde, R.S. (2009). Functional depletion of mahogunin by cytosolically exposed prion protein contributes to neurodegeneration. Cell *137*, 1136–1147.

Chiabudini, M., Tais, A., Zhang, Y., Hayashi, S., Wölfle, T., Fitzke, E., and Rospert, S. (2014). Release factor eRF3 mediates premature translation termination on polylysine-stalled ribosomes in Saccharomyces cerevisiae. Mol. Cell. Biol. *34*, 4062–4076.

Defenouillère, Q., Yao, Y., Mouaikel, J., Namane, A., Galopier, A., Decourty, L., Doyen, A., Malabat, C., Saveanu, C., Jacquier, A., et al. (2013). Cdc48-associated complex bound to 60S particles is required for the clearance of aberrant translation products. Proc. Natl. Acad. Sci. USA *110*, 5046–5051.

Doma, M.K., and Parker, R. (2006). Endonucleolytic cleavage of eukaryotic mRNAs with stalls in translation elongation. Nature *440*, 561–564.

Flicek, P., Amode, M.R., Barrell, D., Beal, K., Billis, K., Brent, S., Carvalho-Silva, D., Clapham, P., Coates, G., Fitzgerald, S., et al. (2014). Ensembl 2014. Nucleic Acids Res. *42*, D749–D755.

Higgins, R., Gendron, J.M., Rising, L., Mak, R., Webb, K., Kaiser, S.E., Zuzow, N., Riviere, P., Yang, B., Fenech, E., et al. (2015). The unfolded protein response triggers site-specific regulatory ubiquitylation of 40S ribosomal proteins. Mol. Cell *59*, 35–49.

Ito-Harashima, S., Kuroha, K., Tatematsu, T., and Inada, T. (2007). Translation of the poly(A) tail plays crucial roles in nonstop mRNA surveillance via translation repression and protein destabilization by proteasome in yeast. Genes Dev. *21*, 519–524.

Kaida, D., Berg, M.G., Younis, I., Kasim, M., Singh, L.N., Wan, L., and Dreyfuss, G. (2010). U1 snRNP protects pre-mRNAs from premature cleavage and polyadenylation. Nature *468*, 664–668.

Koutmou, K.S., Schuller, A.P., Brunelle, J.L., Radhakrishnan, A., Djuranovic, S., and Green, R. (2015). Ribosomes slide on lysine-encoding homopolymeric A stretches. eLife *4*, 4.

Kuroha, K., Akamatsu, M., Dimitrova, L., Ito, T., Kato, Y., Shirahige, K., and Inada, T. (2010). Receptor for activated C kinase 1 stimulates nascent polypeptide-dependent translation arrest. EMBO Rep. *11*, 956–961.

Kwon, I., Xiang, S., Kato, M., Wu, L., Theodoropoulos, P., Wang, T., Kim, J., Yun, J., Xie, Y., and McKnight, S.L. (2014). Poly-dipeptides encoded by the C9orf72 repeats bind nucleoli, impede RNA biogenesis, and kill cells. Science *345*, 1139–1145.

Letzring, D.P., Wolf, A.S., Brule, C.E., and Grayhack, E.J. (2013). Translation of CGA codon repeats in yeast involves quality control components and ribosomal protein L1. RNA *19*, 1208–1217.

Lin, Y.J., Huang, L.H., and Huang, C.T. (2013). Enhancement of heterologous gene expression in Flammulina velutipes using polycistronic vectors containing a viral 2A cleavage sequence. PLoS ONE *8*, e59099.

Lu, J., and Deutsch, C. (2008). Electrostatics in the ribosomal tunnel modulate chain elongation rates. J. Mol. Biol. *384*, 73–86.

Lyumkis, D., Oliveira dos Passos, D., Tahara, E.B., Webb, K., Bennett, E.J., Vinterbo, S., Potter, C.S., Carragher, B., and Joazeiro, C.A.P. (2014). Structural basis for translational surveillance by the large ribosomal subunit-associated protein quality control complex. Proc. Natl. Acad. Sci. USA *111*, 15981–15986.

Mizielinska, S., Grönke, S., Niccoli, T., Ridler, C.E., Clayton, E.L., Devoy, A., Moens, T., Norona, F.E., Woollacott, I.O.C., Pietrzyk, J., et al. (2014). C9orf72 repeat expansions cause neurodegeneration in Drosophila through arginine-rich proteins. Science *345*, 1192–1194.

Pisareva, V.P., Skabkin, M.A., Hellen, C.U.T., Pestova, T.V., and Pisarev, A.V. (2011). Dissociation by Pelota, Hbs1 and ABCE1 of mammalian vacant 80S ribosomes and stalled elongation complexes. EMBO J. *30*, 1804–1817.

Presnyak, V., Alhusaini, N., Chen, Y.H., Martin, S., Morris, N., Kline, N., Olson, S., Weinberg, D., Baker, K.E., Graveley, B.R., and Coller, J. (2015). Codon optimality is a major determinant of mRNA stability. Cell *160*, 1111–1124.

Ran, F.A., Hsu, P.D., Wright, J., Agarwala, V., Scott, D.A., and Zhang, F. (2013). Genome engineering using the CRISPR-Cas9 system. Nat. Protoc. *8*, 2281–2308.

CellPress

Saito, K., Horikawa, W., and Ito, K. (2015). Inhibiting K63 polyubiquitination abolishes no-go type stalled translation surveillance in Saccharomyces cerevisiae. PLoS Genet. *11*, e1005197.

Shao, S., and Hegde, R.S. (2014). Reconstitution of a minimal ribosome-associated ubiquitination pathway with purified factors. Mol. Cell *55*, 880–890.

Shao, S., von der Malsburg, K., and Hegde, R.S. (2013). Listerin-dependent nascent protein ubiquitination relies on ribosome subunit dissociation. Mol. Cell *50*, 637–648.

Shao, S., Brown, A., Santhanam, B., and Hegde, R.S. (2015). Structure and assembly pathway of the ribosome quality control complex. Mol. Cell *57*, 433–444.

Shcherbik, N., Chernova, T.A., Chernoff, Y.O., and Pestov, D.G. (2016). Distinct types of translation termination generate substrates for ribosome-associated quality control. Nucleic Acids Res. *44*, 6840–6852.

Shen, P.S., Park, J., Qin, Y., Li, X., Parsawar, K., Larson, M.H., Cox, J., Cheng, Y., Lambowitz, A.M., Weissman, J.S., et al. (2015). Protein synthesis. Rqc2p and 60S ribosomal subunits mediate mRNA-independent elongation of nascent chains. Science *347*, 75–78.

Shoemaker, C.J., and Green, R. (2011). Kinetic analysis reveals the ordered coupling of translation termination and ribosome recycling in yeast. Proc. Natl. Acad. Sci. USA *108*, E1392–E1398.

Shoemaker, C.J., and Green, R. (2012). Translation drives mRNA quality control. Nat. Struct. Mol. Biol. *19*, 594–601.

Shoemaker, C.J., Eyler, D.E., and Green, R. (2010). Dom34:Hbs1 promotes subunit dissociation and peptidyl-tRNA drop-off to initiate no-go decay. Science *330*, 369–372.

Subtelny, A.O., Eichhorn, S.W., Chen, G.R., Sive, H., and Bartel, D.P. (2014). Poly(A)-tail profiling reveals an embryonic switch in translational control. Nature *508*, 66–71.

Tsuboi, T., Kuroha, K., Kudo, K., Makino, S., Inoue, E., Kashima, I., and Inada, T. (2012). Dom34:hbs1 plays a general role in quality-control systems by dissociation of a stalled ribosome at the 3′ end of aberrant mRNA. Mol. Cell *46*, 518–529.

Verma, R., Oania, R.S., Kolawa, N.J., and Deshaies, R.J. (2013). Cdc48/p97 promotes degradation of aberrant nascent polypeptides bound to the ribosome. eLife *2*, e00308.

Wang, F., Canadeo, L.A., and Huibregtse, J.M. (2015). Ubiquitination of newly synthesized proteins at the ribosome. Biochimie *114*, 127–133.

Yonashiro, R., Tahara, E.B., Bengtson, M.H., Khokhrina, M., Lorenz, H., Chen, K.C., Kigoshi-Tansho, Y., Savas, J.N., Yates, J.R., Kay, S.A., et al. (2016). The Rqc2/Tae2 subunit of the ribosome-associated quality control (RQC) complex marks ribosome-stalled nascent polypeptide chains for aggregation. eLife *5*, e11794.

STAR★METHODS

KEY RESOURCES TABLE

REAGENT or RESOURCE	SOURCE	IDENTIFIER
Antibodies		
Rabbit polyclonal anti-ZNF598 (Figure 2A)	GeneTex	Cat. #GTX119245; RRID: AB_10619017
Rabbit polyclonal anti-ZNF598 (Figures 3A and S2C–S3E)	Abcam	Cat. #ab80458; RRID: AB_2221273
Rabbit monoclonal anti-eS10	Abcam	Cat. #ab151550
Rabbit monoclonal anti-uS10	Abcam	Cat. #ab133776
Rabbit polyclonal anti-uS3	Bethyl Labs	Cat. #A303-840A; RRID: AB_2615588
Rabbit polyclonal anti-eS19	Bethyl Labs	Cat. #A304-002A; RRID: AB_2620351
Rabbit polyclonal anti-uS5	Bethyl Labs	Cat. #A303-794A; RRID: AB_11218192
Rabbit polyclonal anti-uS9	Santa Cruz Biotechnology	Cat. #sc-102087; RRID: AB_2269633
Rabbit polyclonal anti-uL6	Santa Cruz Biotechnology	Cat. #sc-102085; RRID: AB_2182219
Rabbit monoclonal anti-uL2	Abcam	Cat. #ab169538
Rabbit monoclonal anti-eS24	Abcam	Cat. #ab196652
Mouse monoclonal anti-HA	Covance Research Products Inc.	Cat. #MMS-101P; RRID: AB_2314672
Mouse monoclonal anti-Flag	Sigma-Aldrich	Cat. #F3165; RRID: AB_259529
Rabbit polyclonal anti-GFP	Chakrabarti and Hegde, 2009	N/A
Rabbit polyclonal anti-RFP	Chakrabarti and Hegde, 2009	N/A
Rabbit polyclonal anti-NEMF	Shao et al., 2015	N/A
HRP conjugated goat anti-rabbit	Jackson Immunoresearch	Cat. #111-035-003; RRID: AB_2313567
HRP conjugated goat anti-mouse	Jackson Immunoresearch	Cat. #115-035-003; RRID: AB_10015289
Chemicals, Peptides, and Recombinant Proteins		
3× Flag peptide	Sigma-Aldrich	Cat. #F4799
Anti-Flag M2 affinity resin	Sigma-Aldrich	Cat. #A2220
Ni-NTA agarose	QIAGEN	Cat. #30210
Complete protease inhibitor cocktail, EDTA-free	Roche	Cat. #11 873 580 001
Cycloheximide	Sigma-Aldrich	Cat. #C4859; CAS: 66-81-9
Hygromycin B	Millipore	Cat. #400051-100KU; CAS: 31282-04-9
Blasticidin S	Santa Cruz Biotechnology	Cat. #sc204655; CAS: 3513-03-9
MG132	Cayman Chemical	Cat. #10012628; CAS: 133407-82-6
Doxycycline	Sigma-Aldrich	Cat. #D9891; CAS: 24390-14-5
Creatine phosphate	Roche	Cat. #621714
Creatine kinase	Roche	Cat. #127566
ZNF598-TEV-3xFlag (human)	This study	N/A
His-Ubiquitin	Boston Biochem	Cat. #U-530
HA-Ubiquitin	Boston Biochem	Cat. #U-110
Methylated Ubiquitin	Boston Biochem	Cat. #U-501
UbcH5a	Boston Biochem	Cat. #E2-616
GST-UBE1 (human)	Boston Biochem	Cat. #E-306
USP2-CD	Boston Biochem	Cat. #E-504
Experimental Models: Cell Lines		
HEK293T	ATCC	CRL-3216
Flp-In T-REx 293	Thermo Fisher	Cat. #R78007
Recombinant DNA		
pcDNA3.1 ZNF598-TEV-3xFlag	This study	N/A
pcDNA3.1 ΔRING-ZNF598-TEV-3xFlag	This study	N/A

(*Continued on next page*)

Continued

REAGENT or RESOURCE	SOURCE	IDENTIFIER
pmGFP-P2A-K_0-P2A-RFP	This study	N/A
pmGFP-P2A-SL-P2A-RFP	This study	N/A
pmGFP-P2A-$(K^{AAA})_{12}$-P2A-RFP	This study	N/A
pmGFP-P2A-$(K^{AAA})_{20}$-P2A-RFP	This study	N/A
pmGFP-P2A-$(K^{AAG})_{12}$-P2A-RFP	This study	N/A
pmGFP-P2A-$(K^{AAG})_{20}$-P2A-RFP	This study	N/A
pmGFP-P2A-$(R^{CGA})_{10}$-P2A-RFP	This study	N/A
pmGFP-P2A-$(R^{CGA})_{20}$-P2A-RFP	This study	N/A
pmGFP-P2A-$(R^{CGG})_{20}$-P2A-RFP	This study	N/A
pcDNA 5/FRT/TO-GFP-P2A-$(K^{AAA})_{21}$-P2A-RFP	This study	N/A
pcDNA 5/FRT/TO-GFP-P2A-$(K)_0$-P2A-RFP	This study	N/A
pcDNA 5/FRT/TO-eS10-HA	This study	N/A
pcDNA 5/FRT/TO-eS10-K139R-HA	This study	N/A
pcDNA 5/FRT/TO-eS10-K138R/K139R-HA	This study	N/A
pcDNA 5/FRT/TO-uS10-HA	This study	N/A
pcDNA 5/FRT/TO-uS10-K8R-HA	This study	N/A
pcDNA 5/FRT/TO-uS10-K4R/K8R-HA	This study	N/A
pOG44 Flp-Recombinase Expression Vector	Thermo Fisher	Cat. #V600520
pX330-U6-Chimeric_BB-CBh-hSpCas9	Ran et al., 2013	Addgene Plasmid #42230
Sequence-Based Reagents		
Silencer Select Pre-designed siRNA against ZNF598	Life Technologies	Cat. #4392420; siRNA ID #s40509
Silencer Select Pre-designed siRNA against NEMF	Life Technologies	Cat. #4392420; siRNA ID #s17483
Silencer Select Pre-designed siRNA negative control #1	Life Technologies	Cat. #4392420
guide RNA targeting exon 1 of ZNF598 gene 5′-TAGAGCAGCGGTAGCACACC-3′	This study	N/A
Nucleotide sequence of $(K)_0$ insert: CGCCATGGCGACCCCCGGGGGATCC	This study	N/A
Nucleotide sequence of $(K^{AAA})_n$ inserts: CGCCATGGCGACC$(AAA)_n$CCCGGGGGATCC	This study	N/A
Nucleotide sequence of $(K^{AAG})_n$ inserts: CGCCATGGCGACC$(AAG)_n$CCCGGGGGATCC	This study	N/A
Nucleotide sequence of $(R^{CGA})_n$ inserts: CGCCATGGCGACC$(CGA)_n$CCCGGGGGATCC	This study	N/A
Nucleotide sequence of $(R^{CGG})_n$ inserts: CGCCATGGCGACC$(CGG)_n$CCCGGGGGATCC	This study	N/A
Nucleotide sequence of SL insert: CGCCCTGTTCCACTATAGGGCACCTCCCCGC GCACCACCGCCGACGTCGGCGGTGGTGCGC GGGGAGGTGCCCTATAGCGGTAC	This study	N/A
Software and Algorithms		
Ensembl BioMart tool (online version)	Flicek et al., 2014	http://www.ensembl.org/biomart
FlowJo 10.1r5	FlowJo, LLC	https://www.flowjo.com/
GraphPad Prism 6.05	GraphPad Software	http://graphpad.com/
Other		
TransIt 293	Mirus	Cat. #MIR 2705
Lipofectamine RNAiMAX	Thermo Fisher	Cat. #13778150
SuperSignal West Pico Chemiluminescent substrate	Thermo Fisher	Cat. #34080
Rabbit reticulocyte lysate (RRL)	Green Hectares	N/A
DMEM, high glucose, GlutaMAX Supplement, pyruvate	Thermo Fisher	Cat. #10569010
Tetracycline-free fetal calf serum (FCS)	BioSera	Cat. #FB-1001T/500

CONTACT FOR REAGENT AND RESOURCE SHARING

Requests for reagents may be directed to Lead Contact Ramanujan S. Hegde (rhegde@mrc-lmb.cam.ac.uk).

EXPERIMENTAL MODEL AND SUBJECT DETAILS

Cell Lines

HEK293T cells were cultured in Dulbecco's Modified Eagle's Medium (DMEM) with 10% fetal calf serum (FCS). Flp-In 293 T-Rex cells were maintained in DMEM with 10% tetracycline-free FCS in the presence of 15 μg/ml blasticidin and 100 μg/ml hygromycin.

METHOD DETAILS

Constructs

Reporter constructs for transient expression were generated starting with the mGFP-N1 vector (Clontech). For generation of stable cell lines, reporter cassettes were sub-cloned into the pcDNA 5/FRT/TO vector. For the nucleotide sequences of the inserts of the various constructs see also Key Resources Table. ZNF598 with a C-terminal 3X-FLAG tag was in the pcDNA3.1 vector. CRISPR-Cas9 mediated ZNF598 knock out used a guide RNA targeting exon 1 of ZNF598 (5′-TAGAGCAGCGGTAGCACACC −3′ designed with CRISPR design tool at crispr.mit.edu) in the pX330-U6 plasmid (Ran et al., 2013).

Cell Cultures

Stable cell lines were generated using the Flp-In system (Invitrogen) according to manufacturer's protocol. CRISPR-Cas9 mediated gene disruption was performed in the stable cell lines harboring $(K^{AAA})_{21}$ reporter cassette as described (Ran et al., 2013): cells were transiently transfected with the pX330 plasmid encoding the gRNA targeting exon 1 of ZNF598 gene. After 3 days, cells were trypsinized and re-plated in 96-well plates at a density of 0.5 cells per well to obtain single cell clones. After 2 weeks of culture, individual clones were expanded and screened for the presence of ZNF598 protein by western-blotting relative to serial dilutions of the starting cell line. Cell lines showing no detectable signal even with over-exposure of the immunoblot were selected for further study. Expression of transgene was induced with doxycycline (1 μg/ml) for 24-48h. Cycloxehimide (100 μg/ml) and DTT (1 mM) treatments were performed in standard medium for 2h. MG132 (20 μM) treatment, where indicated, was for 4h. Transient transfections were performed using TransIt-293 (Mirus) according to manufacturers protocol. siRNA silencing was performed with Lipofectamine RNAiMAX reagent (Life Technologies) using standard methods.

Flow Cytometry Analysis

Trypsinized cells were sedimented (1000 rpm for 5 min at room temperature), resuspended in 10% FCS in PBS, and analyzed using the Becton Dickinson LSR II and FlowJo software.

Western Blot Analysis

Cells were washed with PBS twice and lysed with 100 mM Tris pH 8.0 with 1% SDS then boiled for 10 min with vortexting to shear genomic DNA. 5X SDS-PAGE sample buffer was added for a final concentration of 50 mM Tris, 1%SDS, 10% glycerol, and 100 mM DTT. Samples were analyzed using 10% Tris-Tricine based gels, and transferred to 0.2 μm nitrocellulose membrane (Biorad). Primary antibody incubations were either for 1h at room temperature or 4°C overnight. Detection used HRP-conjugated secondary antibodies and SuperSignal West Pico Chemiluminescent substrate (Thermo Fisher).

Ribosomes Purification

Ribosomes were sedimented from 25 mL of nuclease treated rabbit reticulocyte lysate (RRL; Green Hectares) in a TLA100.4 rotor at 100,000 rpm for 40 min at 4°C. The pellets were briefly washed with Ribosome Wash Buffer (RWB) (20 mM HEPES pH 7.5, 100 mM KAc, 1.5 mM $MgAc_2$, 0.1 mM EDTA), resuspended in a total volume of ~8 mL RWB and gently homogenized in a glass dounce with Teflon pestle. 2 mL aliquots of resuspended ribosomes were layered over a 1 mL sucrose cushion (RWB with 1 M sucrose and 1 mM DTT) and centrifuged using a TLA100.4 rotor at 100,000 rpm at 4°C for 1h. Ribosome pellets were resuspended in a total volume of ~2.2 mls of 20 mM HEPES pH 7.4, 100 mM KAc, 1.5 mM $MgAc_2$, 10% glycerol. The purified ribosomes were frozen in liquid nitrogen and stored at - 80°C. Concentration was measured using absorbance at 260 nm, assuming that1 A_{260} unit corresponds to 16 nM ribosomes.

Recombinant ZNF598-3X-FLAG Protein Purification

C-terminal 3X-Flag-tagged ZNF598 was transfected into HEK293T cells using TransIT 293 (Mirus), and purified after two days of expression. Sixteen 10 cm dishes of confluent ZNF598-expressing cells were harvested in ice cold PBS, sedimented, and lysed in ~1 mL 50 mM HEPES, pH 7.4, 100 mM KAc, 5 mM $MgAc_2$, 100 μg/mL digitonin, 1 mM DTT, 1X EDTA-free Complete protease inhibitor cocktail (Roche) for 20 min on ice. The lysate was clarified in a tabletop microcentrifuge at 4°C for 10 min and incubated with 100 μL of packed anti-Flag affinity resin (Sigma) for 1-1.5 hr at 4°C. The resin was washed three times in 1 mL lysis buffer, three

times in 1 mL 50 mM HEPES, pH 7.4, 400 mM KAc, 5 mM $MgAc_2$, 100 μg/mL digitonin, 1 mM DTT buffer, and three times in 50 mM HEPES, pH 7.4, 100 mM KAc, 5 mM $MgAc_2$ buffer. Elutions were carried out with one column volume of 0.2 mg/ml 3X-Flag peptide in the final wash buffer at room temperature for 30 min. Two sequential elutions were combined to form the final fraction.

In Vitro Ubiquitination of Ribosomes

Ubiquitination reactions contained 1 mM ATP, 10 mM creatine phosphate, 40 ng/ml creatine kinase, 10 μM His-Ubiquitin (Boston Biochem), 100 nM rhGST-UBE1 (Boston Biochem), 200 nM UbcH5a (Boston Biochem), 1.6 to 100 nM ZNF598 (as indicated in individual figure legends), and 220 nM ribosomes in PS buffer (100 mM KAc, 50 mM HEPES, pH 7.4, 5 mM $MgAc_2$). All components except ribosomes and ZNF598 were pre-assembled on ice and incubated at room temperature for 15 min before adding ribosomes and ZNF598. Reactions were for 1h at 32°C. The samples were either analyzed directly, or purified as follows for downstream mass spectrometry analysis. 200 μL reactions were chilled on ice and layered onto 2 mL 10%–50% sucrose gradients in PS buffer. After centrifugation at 55,000 rpm for 1 hr in a TLS-55 rotor (Beckman), eleven 200 μL fractions were removed from the top. The ribosomal fractions (4-11) were pooled (1.6 ml), mixed with 400 ul of 500 mM Tris pH 8.0, 5% SDS, and denatured by heating to 95°C for 5 min. The sample was cooled, diluted with 8 mL of Triton buffer (PBS containing 0.5% Triton X-100, 250 mM NaCl, 20 mM imidazole), and incubated with 20 μL of Ni-NTA-agarose resin overnight with end-over-end rolling at 4°C. The resin was washed 3 times with 1 mL of Triton buffer, and eluted with 2.5x SDS-PAGE sample buffer containing 0.05 M EDTA (for direct SDS-PAGE analysis and mass spectrometry) or PBS with 0.05 M EDTA for deubiquitination with 10 μM USP2 catalytic domain for 1h at 32°C.

Sucrose Gradient Fractionation

Analytical scale 0.2 mL gradients were prepared in 7 × 20 mm centrifuge tubes (Beckman 343775) by successively layering 40 μL of 50%, 40%, 30%, 20%, and 10% sucrose (w/v) in PS buffer. Gradients were then allowed to stand for 1-2 hr at 4°C. In vitro ubiquitination reactions (20 μl) were loaded on top of the gradients, and the samples centrifuged in a TLS- 55 rotor at 55,000 rpm for 30 min at 4°C with the slowest acceleration and deceleration settings. Eleven 20 μL fractions were successively collected from the top and used directly for western blot analysis. Where needed, 10-fold larger gradients were prepared in exactly the same way using 11 × 34 mm tubes (Seton 5011), but centrifuged at 55,000 rpm in the TLS-55 rotor for 1 hr.

Cytosol Fractionation

Actively growing HEK293 T-Rex cells (~80% confluent) expressing eS10-K138/139R-HA (or other ribosomal protein variants) in 6-well plates were washed in PBS and the cytosol extracted with digitonin-containing buffer (25 mM HEPES, pH 7.4, 125 mM KAc, 15 mM $MgAc_2$, 100 μg/mL digitonin, 40U/mL RNase inhibitor, 50 μg/mL CHX, 1 mM DTT, 1X protease inhibitor cocktail) on ice for 5 min. The lysate was spun at maximum speed in a benchtop microcentrifuge for 10 min at 4°C, and the supernatant was separated on a 2 mL 10%–50% sucrose gradient as detailed above. Fractions were collected and subjected to TCA precipitation for analysis by immunoblotting.

QUANTIFICATION AND STATISTICAL ANALYSIS

In Figure 1B, the quantitative value that is graphed represents the median of the RFP:GFP ratio for 20,000 GFP positive events normalized to the value observed for the control construct [$(K)_0$]. Error bars represent the "robust SD" value generated in FlowJo, and represents 68.26% of the events around the median. All experiments were repeated at least twice, and a representative result is shown. No statistical analyses were performed in this study.

Molecular Cell, Volume *65*

Supplemental Information

Initiation of Quality Control during Poly(A) Translation Requires Site-Specific Ribosome Ubiquitination

Szymon Juszkiewicz and Ramanujan S. Hegde

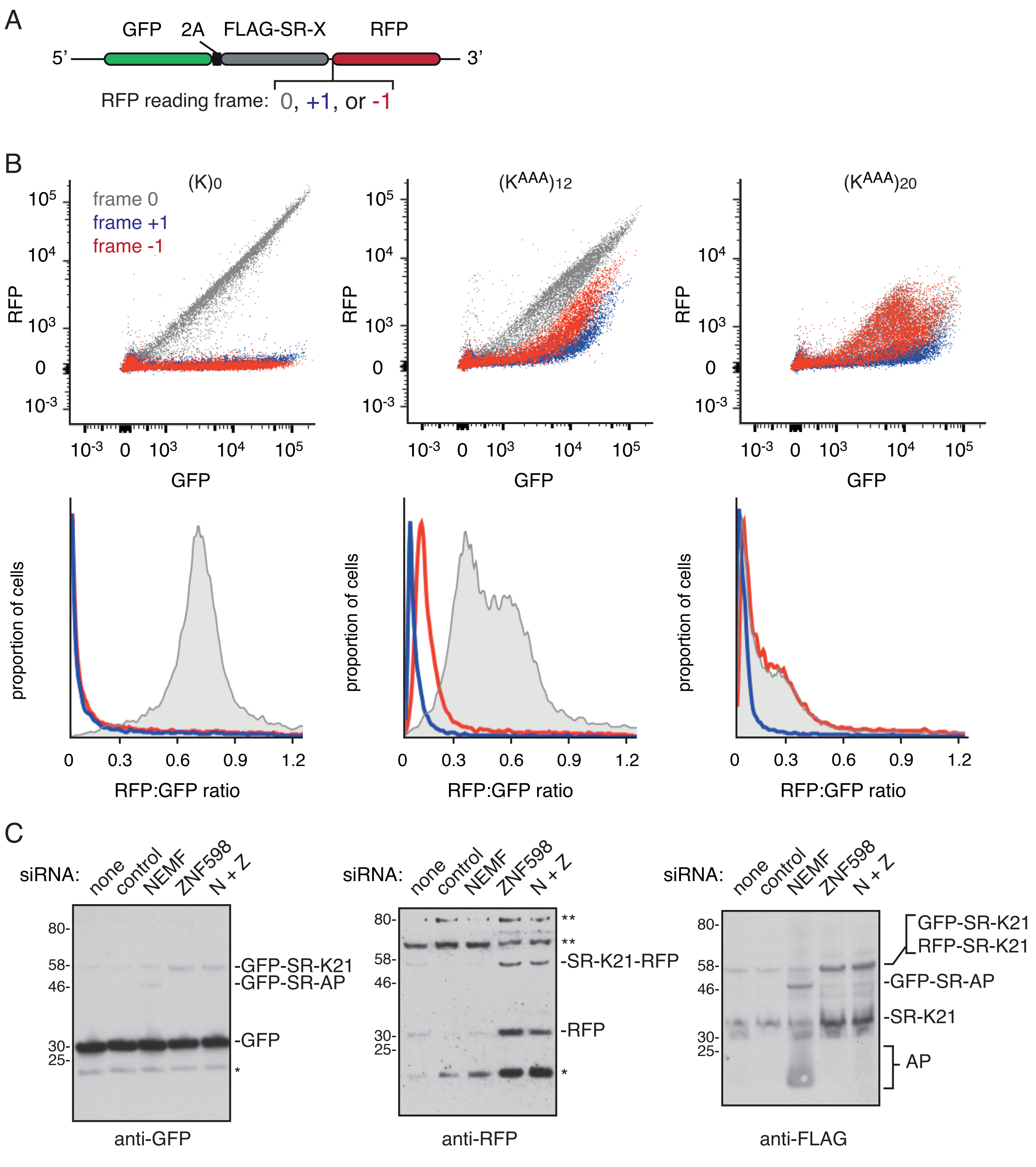
A
GFP
2A
FLAG-SR-X
RFP
5'
3'
RFP reading frame: 0, +1, or -1
B
(K)0
(KAAA)12
(KAAA)20
frame 0
frame +1
frame -1
RFP
GFP
proportion of cells
RFP:GFP ratio
C
siRNA:
none
control
NEMF
ZNF598
N + Z
-GFP-SR-K21
-GFP-SR-AP
-GFP
-SR-K21-RFP
-RFP
GFP-SR-K21
RFP-SR-K21
-GFP-SR-AP
-SR-K21
AP
anti-GFP
anti-RFP
anti-FLAG

Fig. S1. Characterization of the stalling reporter (related to Fig. 1).
(**A**) Schematic representation of constructs used for monitoring frameshifting. Single nucleotide insertion or deletion was introduced just preceding RFP to change its reading frame relative to the remainder of the construct. (**B**) HEK293 cells were transfected with either $(K)_0$, $(K^{AAA})_{12}$ or $(K^{AAA})_{20}$ constructs in each of the three RFP reading frames and analysed by flow cytometry after 24 hours. Shown below each scatter plot of GFP and RFP levels are the corresponding histograms of the RFP:GFP ratio. Grey is the native frame where RFP is in frame with GFP. Red and blue are constructs with RFP in the -1 and +1 frames, respectively. Note that with $(K)_0$, RFP signal is only seen when it is in the native frame. By contrast, RFP signal is detectable in each reading frame for the $(K^{AAA})_{12}$ and $(K^{AAA})_{20}$ constructs, with a slightly lower preference for the +1 frame. The RFP:GFP ratio observed at the peak of each histogram was used as an indicator of read-through into that frame. The sum of the values for all reading frames was used to estimate the net read-through (i.e., the RFP:GFP ratio that would have been seen if there was no frameshifting). This analysis shows that the compensated RFP:GFP ratio for $(K^{AAA})_{12}$ is ~88% of that seen for $(K)_0$, indicating modest stalling of ribosomes. The compensated ratio for $(K^{AAA})_{20}$ is ~43% of that seen for $(K)_0$, indicating that most ribosomes stall in this case. (**C**) The full blots for the indicated proteins are shown for cell lysates from a stable cell line expressing $(K^{AAA})_{21}$. The experiment shown is from Fig. 2B. Here, $(K^{AAA})_{21}$ cells were subjected to the indicated siRNA treatment for 72h, reporter expression induced for 24h, and analyzed by immunoblotting. If the P2A peptides work perfectly, GFP, FLAG-SR-K21, and RFP would always be separate protein products. Slight inefficiency leads to fusions of GFP with SR (detectable on both the GFP and FLAG blots), or SR with RFP (detectable on both the FLAG and RFP blots). The triple fusion was not detectable, presumably because the rate of failure at both P2A peptides is exceedingly infrequent. Double-asterisks indicate non-specific bands detected by the anti-RFP antibody. Single asterisks indicate truncated N-terminal fragments of GFP and RFP generated by unknown means, but visible in cells even expressing only these individual proteins. Note that the fusion products behave as expected in cells knocked down for either NEMF, ZNF598, or both. Thus, NEMF knockdown produces the GFP-SR arrest product (AP) visible on the FLAG and GFP blots, while ZNF598 knockdown results in increased GFP-SR-K21 and SR-K21-RFP.

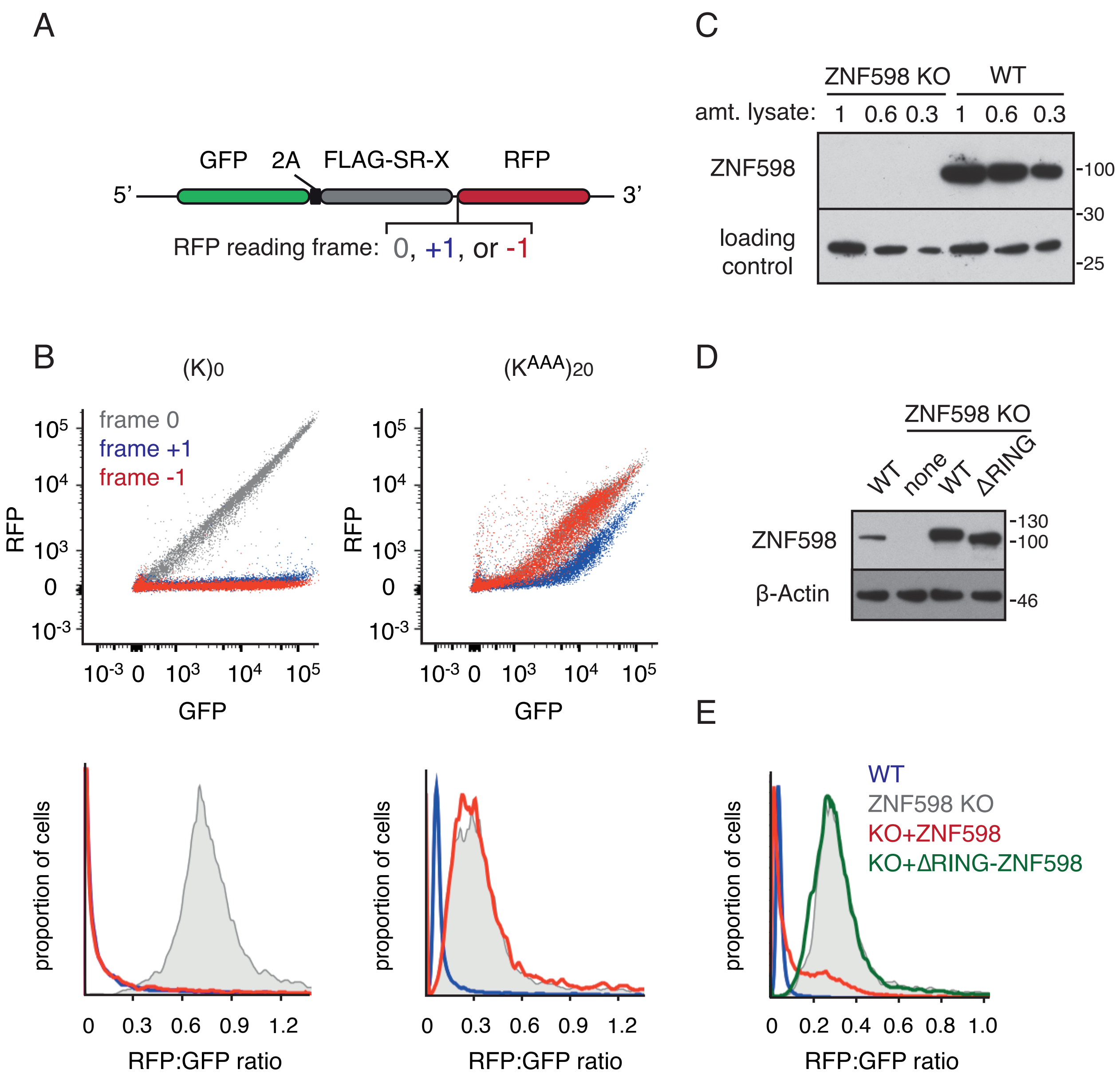

Fig. S2. Characterization of ZNF598 knockout cells (related to Fig. 2).
(**A**) Schematic representation of constructs used for monitoring frameshifting. Single nucleotide insertion or deletion was introduced just preceding RFP to change its reading frame relative to the remainder of the construct. (**B**) ZNF598 KO cells were transfected with either $(K)_0$ or $(K^{AAA})_{20}$ constructs in each of the three RFP reading frames and analysed by flow cytometry after 24h. Shown below each scatter plot of GFP and RFP levels are the corresponding histograms of the RFP:GFP ratio. Grey is the native frame where RFP is in frame with GFP. Red and blue are constructs with RFP in the -1 and +1 frames, respectively. Note that with $(K)_0$, RFP signal is only seen when it is in the native frame. By contrast, RFP signal is seen regardless of reading frame for the $(K^{AAA})_{20}$ construct, with a slightly lower preference for the +1 frame. Thus, the ribosome samples all three reading frames beyond the $(K^{AAA})_{20}$ region, indicative of frameshifting during poly(A) translation. The RFP:GFP ratio observed at the peak of each histogram was used as an indicator of read-through into that frame. The sum of the values for all reading frames was used to estimate the net read-through (i.e., the RFP:GFP ratio that would have been seen if there was no frameshifting). This analysis shows that the compensated RFP:GFP ratio for $(K^{AAA})_{20}$ is ~90% of that seen for $(K)_0$, indicating minimal stalling of ribosomes in ZNF598 knockout cells. (**C**) Serial dilutions of cell lysates from ZNF598 knockout (KO) and wild type (WT) cells were analysed by immunoblotting to confirm the complete absence of ZNF598 protein expression in the KO cells. (**D**) ZNF598 KO cells were transfected with the indicated constructs and analysed by immunoblotting after 24h to verify comparable expression levels of the wild type and ΔRING mutant ZNF598. These samples are from the experiment shown in Fig. 2D. (**E**) Histograms of the RFP:GFP ratio are plotted for the experiment in Fig. 2D relative to wild type cells (blue) analyzed in parallel. The result shows that rescue of the knockout cells with ZNF598 (red) restores the RFP:GFP ratio to wild type levels for the vast majority of cells. By contrast, ΔRING-ZNF598 has no detectable rescue and is identical to the ZNF598 knockout cells (grey versus green) .

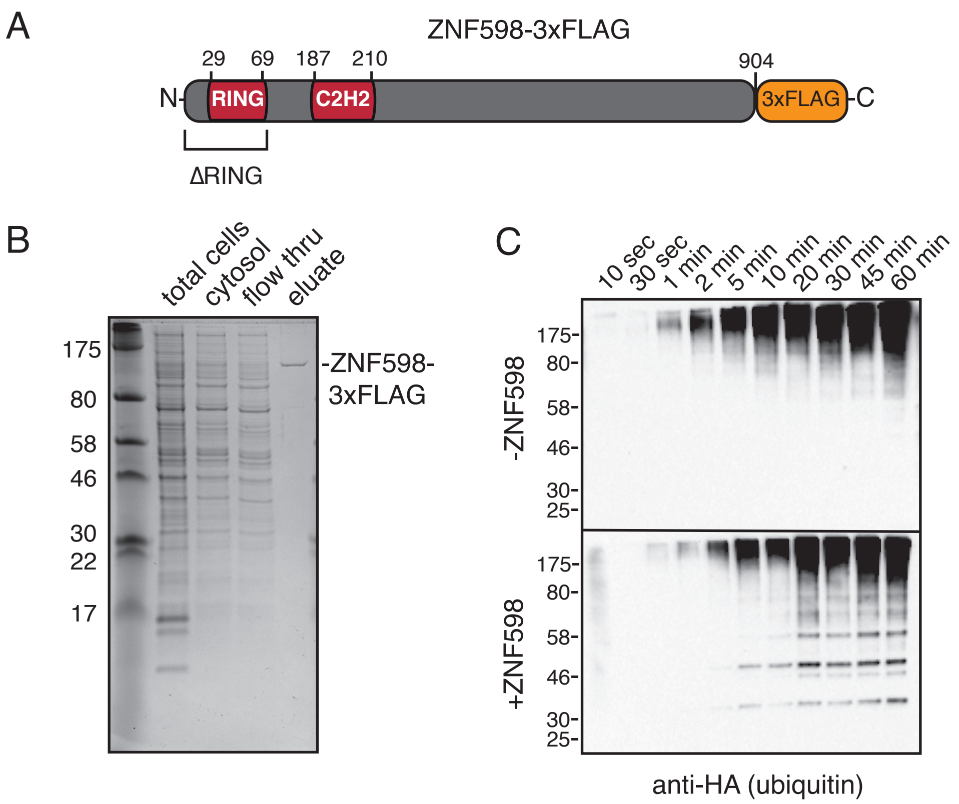

D

total spectral counts

protein	-ZNF598	+ZNF598
eS10	5	50
uS3	4	35
uS10	0	20
eS4	12	19
uS5	7	15
eL13	5	15
eL8	9	14
eS7	5	11
eS6	4	10
eS1	4	9
uL30	10	9
eL24	6	9
eL19	6	9
eL6	3	7
eEF1	4	7
eS8	4	6
uL2	7	6
uL3	5	6
eL14	4	6
uL5	5	5

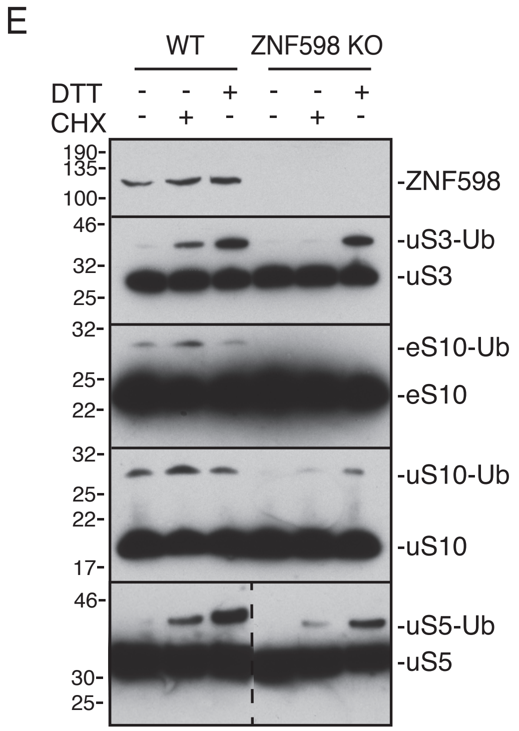

Fig. S3. Characterization of ribosomal protein ubiquitination (related to Fig. 3).
(**A**) Schematic representation of ZNF598 constructs used in this study illustrating the position of the affinity tag used for purification, and the region deleted to generate a ligase-inactive version. (**B**) Coomasie stained gel of fractions from a typical 3xFLAG-ZNF598 purification from transiently transfected cells. (**C**) Time course of in vitro ubiquitination of ribosomes without or with 50 nM ZNF598. The reaction included HA-ubiquitin, and the blot was probed with anti-HA. (**D**) Results of the mass spectrometry analysis of ubiquitinated proteins recovered from the experiment illustrated in Fig. 3B. Proteins identified in red showed a clear (more than five-fold) enrichment in the sample containing ZNF598. (**E**) Wild type (WT) and ZNF598 knockout (KO) cells were treated with 100 µg/ml cycloheximide or 1 mM DTT for 2 h as indicated and immunoblotted for the indicated ribosomal proteins to analyze their ubiquitination status. While uS5 was not appreciably affected in its ubiquitination under any condition by the absence of ZNF598, the other proteins were affected under some, but not all conditions. Ubiquitinated eS10 was not detected under any condition in the knockout cells.

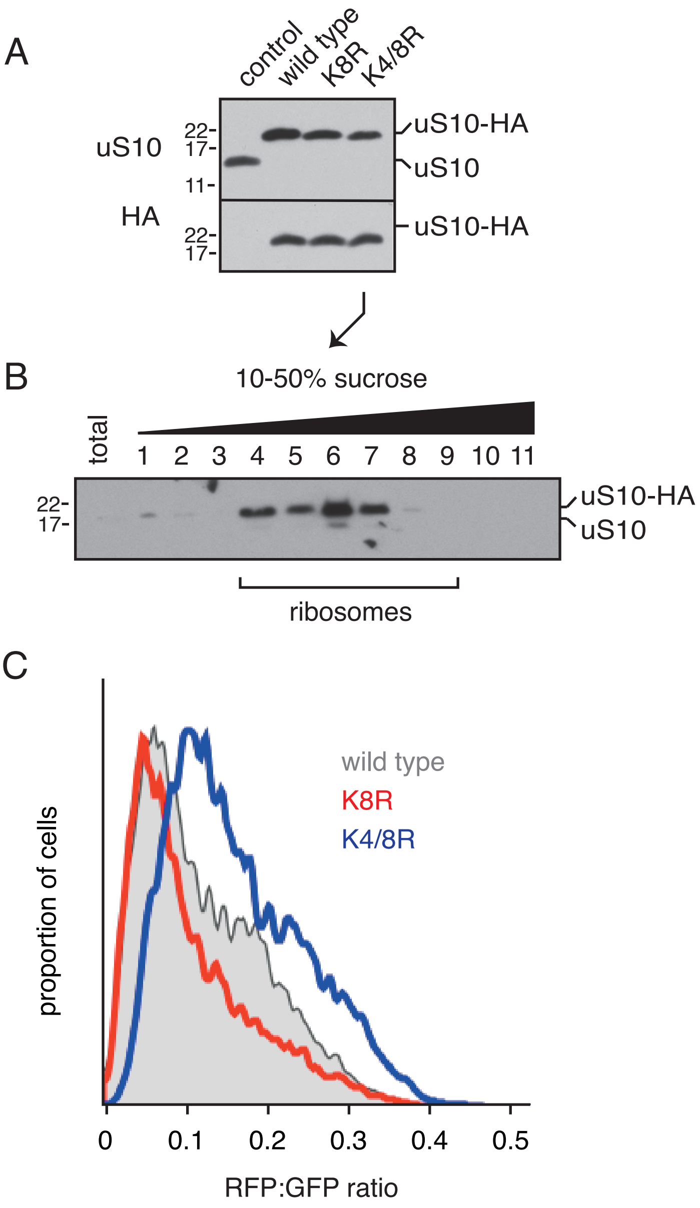

Fig. S4. Analysis of stalling in cells containing mutant uS10 (related to Fig. 4).
(**A**) Cytosol from cells stably expressing different HA-tagged variants of uS10 was analyzed by immunoblotting for uS10 and the HA tag. (**B**) Cytosol from cells stably expressing the HA-tagged K4/8R mutant of uS10 was separated on a 10-50% sucrose gradient and immunoblotted for uS10. The fractions containing ribosomes are indicated. Similar results were seen for other uS10 variants (not shown). (**C**) The indicated HA-uS10 expressing cells were transfected with the $(K^{AAA})_{20}$ reporter and analyzed by flow cytometry 24h later. The RFP:GFP ratio of all transfected cells is shown as a histogram (uS10-HA in grey, uS10-K8R-HA in red, uS10-K4/8R-HA in blue).
